# Epigenetic supersimilarity of monozygotic twin pairs

**DOI:** 10.1186/s13059-017-1374-0

**Published:** 2018-01-09

**Authors:** Timothy E. Van Baak, Cristian Coarfa, Pierre-Antoine Dugué, Giovanni Fiorito, Eleonora Laritsky, Maria S. Baker, Noah J. Kessler, Jianrong Dong, Jack D. Duryea, Matt J. Silver, Ayden Saffari, Andrew M. Prentice, Sophie E. Moore, Akram Ghantous, Michael N. Routledge, Yun Yun Gong, Zdenko Herceg, Paolo Vineis, Gianluca Severi, John L. Hopper, Melissa C. Southey, Graham G. Giles, Roger L. Milne, Robert A. Waterland

**Affiliations:** 1 USDA/ARS Children’s Nutrition Research Center, Department of Pediatrics, Baylor College of Medicine, Houston, TX USA; 2 Department of Molecular and Cellular Biology, Baylor College of Medicine, Houston, TX USA; 3 Cancer Epidemiology and Intelligence Division, Cancer Council Victoria, Melbourne, VIC Australia; 4 Centre for Epidemiology and Biostatistics, Melbourne School for Global and Population Health, University of Melbourne, Melbourne, VIC Australia; 5 Department of Medical Sciences, University of Torino and Italian Institute for Genomic Medicine, Torino, Italy; 6 MRC Unit The Gambia, Keneba, Gambia; 7 MRC International Nutrition Group at LSHTM, London, UK; 8 Division of Women’s Health, King’s College London, London, UK; 9 Epigenetics Group, International Agency for Research on Cancer, Lyon, France; 10 LICAMM, School of Medicine, University of Leeds, Leeds, UK; 11 School of Food Science & Nutrition, University of Leeds, Leeds, UK; 12 MRC-PHE Center for Environment and Health, School of Public Health, Imperial College London, London, UK; 13 Italian Institute for Genomic Medicine, Torino, Italy; 14 CESP Inserm, Facultés de medicine Université Paris-Sud, Paris, France; 15 Genetic Epidemiology Laboratory, Department of Pathology, University of Melbourne, Melbourne, Victoria Australia; 16 Department of Molecular and Human Genetics, Baylor College of Medicine, Houston, TX USA

**Keywords:** Epigenetics, Twins, Monozygotic, Dizygotic, Cancer, Metastable epialleles, Developmental programming, DOHaD

## Abstract

**Background:**

Monozygotic twins have long been studied to estimate heritability and explore epigenetic influences on phenotypic variation. The phenotypic and epigenetic similarities of monozygotic twins have been assumed to be largely due to their genetic identity.

**Results:**

Here, by analyzing data from a genome-scale study of DNA methylation in monozygotic and dizygotic twins, we identified genomic regions at which the epigenetic similarity of monozygotic twins is substantially greater than can be explained by their genetic identity. This “epigenetic supersimilarity” apparently results from locus-specific establishment of epigenotype prior to embryo cleavage during twinning. Epigenetically supersimilar loci exhibit systemic interindividual epigenetic variation and plasticity to periconceptional environment and are enriched in sub-telomeric regions. In case-control studies nested in a prospective cohort, blood DNA methylation at these loci years before diagnosis is associated with risk of developing several types of cancer.

**Conclusions:**

These results establish a link between early embryonic epigenetic development and adult disease. More broadly, epigenetic supersimilarity is a previously unrecognized phenomenon that may contribute to the phenotypic similarity of monozygotic twins.

**Electronic supplementary material:**

The online version of this article (doi:10.1186/s13059-017-1374-0) contains supplementary material, which is available to authorized users.

## Background

Epigenetic mechanisms govern cell type-specific differences in gene expression potential [1]. DNA methylation, which occurs predominantly at CpG dinucleotides in the mammalian genome, is a stable epigenetic mark critical to genomic imprinting, silencing of retrotransposons, and cell type-specific gene expression. Thirty years ago it was proposed that aberrant DNA methylation could function as an “epimutation” and contribute to human disease, analogously to genetic mutations [2]. Indeed, DNA methylation is implicated in cancer [3] and a host of human diseases. Advancing our understanding of the role of DNA methylation in human disease is complicated, however, by the cellular heterogeneity of epigenetic marks, the influence of genetics on epigenetics, and the potential for reverse causality [4, 5].

The characteristics of metastable epialleles (MEs) circumvent these obstacles, offering outstanding opportunities to understand how interindividual epigenetic variation contributes to human disease. MEs are epigenetic variants that are set stochastically in the early embryo and maintained during subsequent cellular differentiation [6]. Consequently, MEs function as epigenetic polymorphisms, i.e., stable and systemic (not cell type-specific) individual variants. Epigenetic metastability was discovered due to visible phenotypic differences among isogenic inbred mice [7]. Interindividual variation in gene expression and phenotype is correlated with stable individual differences in DNA methylation at murine MEs [8].

Reasoning that monozygotic twins offer a human analog of inbred mice, we explored a publicly available genome-scale CpG methylation data set for monozygotic (MZ) and dizygotic (DZ) twins [9] based on the widely utilized Illumina Infinium Human-Methylation 450 (HM450) array. We discovered that many candidate MEs exhibit inordinately high epigenetic similarity in MZ twin pairs—a phenomenon we have termed “epigenetic supersimilarity”. We propose a model to explain this phenomenon, characterize genomic and epigenomic features of epigenetically supersimilar loci and, in a large prospective epidemiologic study, show that methylation at these loci in peripheral blood DNA years before diagnosis is associated with risk of specific types of cancer.

## Results

### Epigenetic supersimilarity in MZ twins

Rather than being predominantly determined by genetics, interindividual variation in DNA methylation at MEs is determined, at least in part, stochastically [6] and influenced by the nutritional milieu of the preimplantation embryo [10–12]. We therefore expected that, at MEs, methylation concordance within MZ twin pairs would be greater than that of unrelated individuals, but comparable to that within DZ twin pairs. To test this, we analyzed a genome-scale DNA methylation data set from Grundberg et al. [9], who used the HM450 array to assess methylation in adipose tissue from adult female twins of European-descent (97 MZ twin pairs and 162 DZ twin pairs). As did Grundberg et al., we discarded low-quality probes potentially affected by single nucleotide polymorphisms (SNPs) and, of the remaining 344,303 probes, focused our analysis on the 10% (34,405) with the highest interindividual variance (hereafter referred to as the top 10%).

**Fig. 1:**
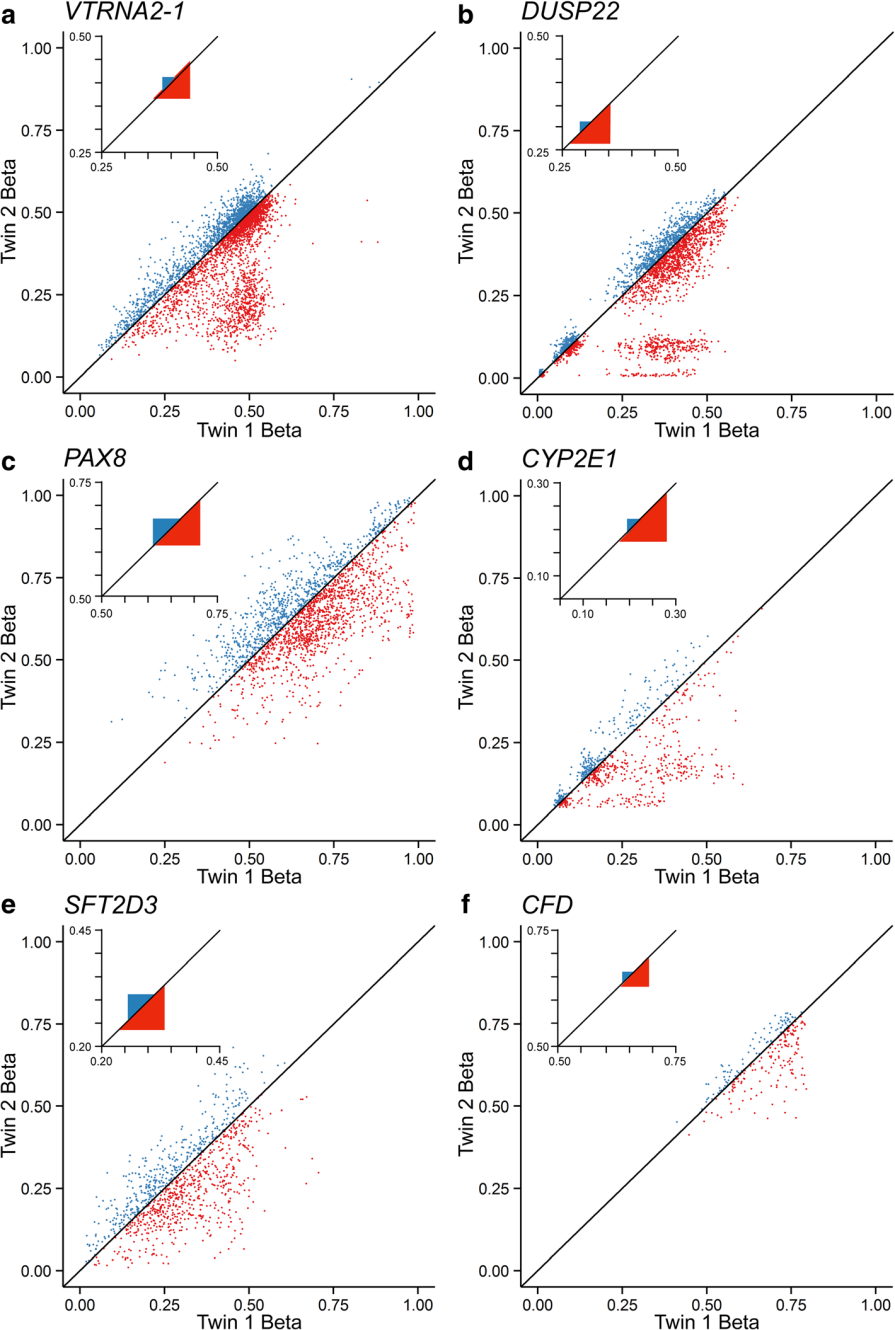
Methylation in MZ twin pairs is highly concordant at candidate metastable epialleles (MEs). Each plot shows probe-specific β values for 97 MZ (*blue*, twin 2 > twin 1) and 162 DZ (*red*, twin 2 < twin 1) twin pairs, at loci previously identified as bona fide or candidate MEs [13, 14]. *Insets* show locus-average mean square errors (MSE) across all the MZ and DZ twins. MSE is much lower in MZ compared to DZ twins. **a** *VTRNA2-1*, 15 probes, 10.6-fold lower MSE (in MZ vs. DZ). **b** *DUSP22*, 11 probes, 16.5-fold lower. **c** *PAX8*, eight probes, 2.5-fold lower. **d** *CYP2E1*, three probes, 10.8-fold lower. **e** *SFT2D3*, four probes, 3.1-fold lower. **f** *CFD*, one probe, 6.6-fold lower

**Fig. 2:**
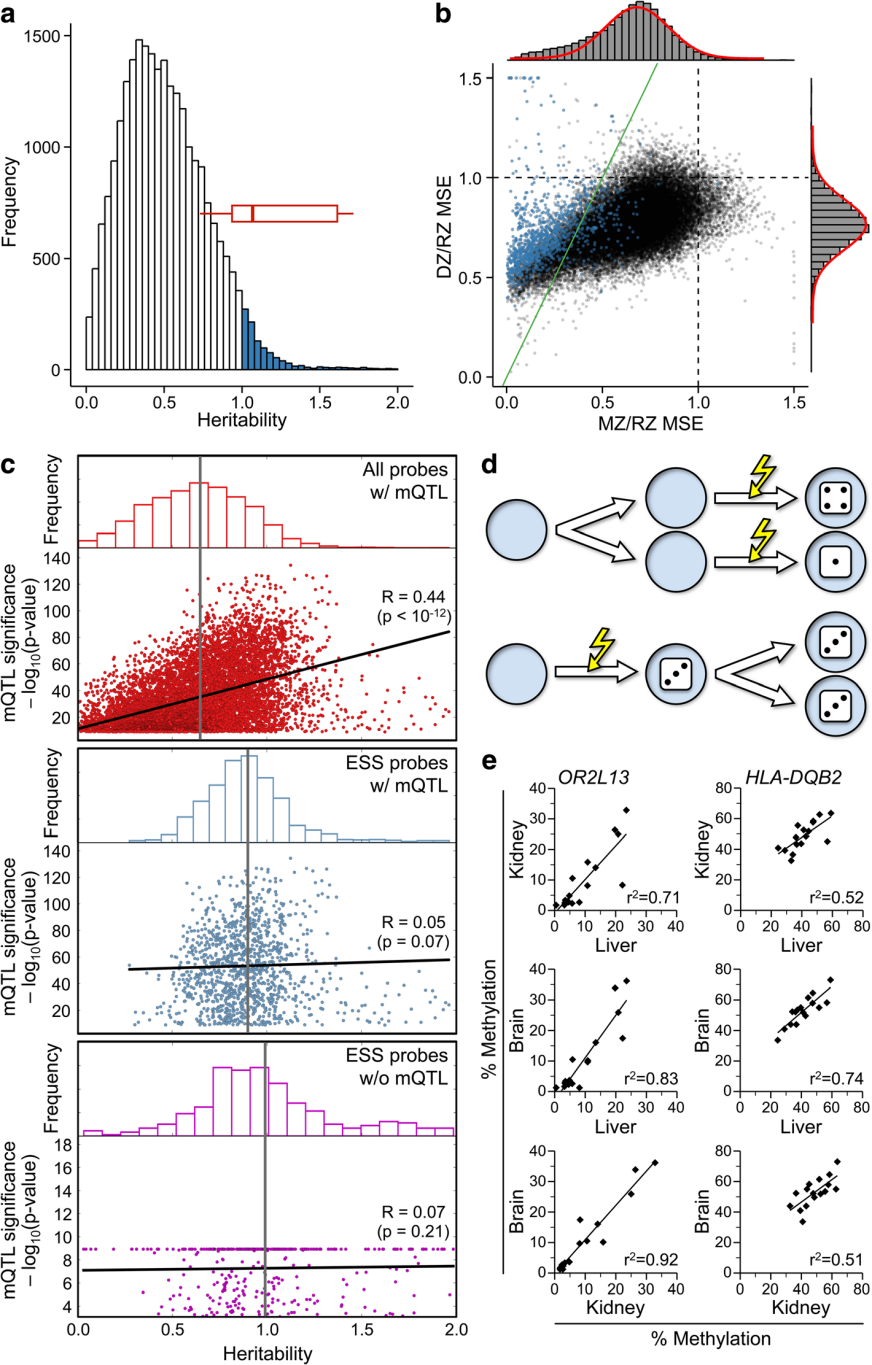
Some HM450 probes exhibit epigenetic supersimilarity (ESS). **a** Distribution of probe-specific narrow-sense heritability (*h*^*2*^) estimates from [9]. (Shown are data on 24,839 probes; 9566 probes with *h*^*2*^ < 0.001 were excluded for clarity.) Of the probes, 1058 show *h*^*2*^ > 1, including most of the probes illustrated in Fig. 1 (*red box plot*). **b** Normalized DZ MSE vs. MZ MSE for the 34,405 probes (top 10%) from Grundberg et al. [9]. Histograms (*right* and *top*) show distribution; *red curves* show best normal fit. Normalized DZ MSE (mean ± standard deviation = 0.76 ± 0.13) is normally distributed, but normalized MZ MSE (0.63 ± 0.23) is skewed left (*P* = 7.0 × 10^–66^). Probes with *h*^*2*^ > 1 are shown in *blue*. Probes to the left of the *green line* (y = 2x) are classified as ESS. **c** Associations between probe-level mQTL and heritability estimates (both from Grundberg et al. [9]). Among the 9708 probes that are both in the top 10% of interindividual variance and positive for mQTL (*top panel*) mean heritability is 0.64 (*gray vertical line*) and positively associated with the strength of mQTL. Among ESS probes positive for mQTL (*middle panel*), mean heritability is 0.90 and not associated with mQTL. Mean heritability of ESS probes negative for mQTL (0.99, *bottom panel*) is similar to that of mQTL-positive ESS probes. **d** Model to explain ESS in MZ twins. Numbers on the dice represent different methylation states at a specific locus. If de novo methylation occurs after embryo cleavage (*top*), each MZ embryo undergoes independent establishment. If de novo methylation occurs prior to embryo cleavage (*bottom*), both MZ embryos inherit the same methylation state. **e** Consistent with this model, bisulfite pyrosequencing in three tissues of 17 cadavers indicates that ESS probes also show systemic interindividual variation. Two examples are shown, *OR2L13* and *HLA-DQB2*

Within regions previously identified as candidate or bona fide MEs [13, 14], we assessed twin–twin methylation concordance inversely by probe-specific mean square error (MSE) of β values. MSE assesses the deviation of a twin pair from the line of identity, providing a direct measure of discordance. Contrary to our expectation, MZ twin concordance in putative ME regions was between 2.5- and 16.5-fold higher than that of DZ twins (Fig. 1). This suggested that establishment of DNA methylation at these regions is under genetic control. To test this, we examined the probe-specific narrow-sense heritability (*h*^*2*^) estimates (based on the ACE method [15]) from Grundberg et al. [9]; *h*^*2*^ is the proportion of phenotypic variation in a population that is attributable to genetic variation [16]. Strikingly, 1058 probes (3% of total) showed *h*^*2*^ estimates > 1 (Fig. 2a). Most of the probes within the candidate MEs featured in Fig. 1 were among them (Fig. 2a), indicating that these superordinate *h*^*2*^ values are not simply a result of sampling error.

Clearly, *h*^*2*^ values > 1 are difficult to interpret. To better understand this observation, we calculated MSE for all 34,405 top 10% probes [9]. To elucidate the extent to which DZ and MZ twins are more similar than pairs of unrelated individuals, probe-specific MSEs were normalized relative to randomized pairs (RZ), simulating pairwise MSE within the general population. DZ/RZ MSE and MZ/RZ MSE were generally < 1, as expected (Fig. 2b). Genetic influences on CpG methylation generally occur when the local sequence context in *cis* (i.e., a haplotype) affects establishment of methylation [17]. Given that DZ twins are identical by descent at 50% of haplotypes [18] and MZ twins at 100% of haplotypes, a model based on genetic determination predicts that the mean normalized DZ MSE should be no more than twice the mean normalized MZ MSE. Hence, for probes to the left of the green line (y = 2x) in Fig. 2b, MZ twin pairs show greater-than-expected similarity in DNA methylation. We refer to this phenomenon as “epigenetic supersimilarity” (ESS). According to the central limit theorem, assuming that probe-specific methylation is determined by many unobserved (genetic) factors, the mean intra-pair errors should be normally distributed. Indeed, normalized DZ MSE are, but normalized MZ MSE are skewed to the left (*P* = 7.0 × 10^–66^) (Fig. 2b). Each probe with DZ/MZ MSE > 2 (corresponding to those left of the green line in Fig. 2b) is > 5 standard deviations (sd) away from the expected normal mean (*P* < 0.0001) (Additional file 1: Figure S1a), well beyond the range of sampling error. Most of the probes for which Grundberg et al. estimated *h*^*2*^ > 1 are characterized as ESS (Fig. 2b). Our initial validation studies found that many ESS probes with interindividual β range < 0.4 in the Grundberg et al. data set [9] are essentially unmethylated in several human primary tissues. We therefore refined the selection criteria to MSE DZ/MZ > 2 *and* an interindividual β range > 0.4, identifying 1580 probes (4.6% of the 34,405) as ESS (Additional file 1: Figure S1b and Additional file 2: Table S1). Across all probes with β range > 0.4, normalized DZ MSE remained normally distributed, but normalized MZ MSE were shifted even further to the left (Additional file 1: Figures S1b, c).

To further test whether the superordinate heritability estimates of Grundberg et al. might somehow result from the genetic identity of MZ twin pairs, we analyzed their data on methylation quantitative trait loci (mQTL), i.e., sequence variants correlated with methylation at specific CpG sites [19]. Grundberg et al. [9] combined their genotyping and HM450 data on 603 adipose tissue samples and applied a conservative significance threshold (*P* < 1.2 × 10^–9^), identifying 9708 mQTL probes within the top 10% of interindividual variance. Among these, as expected, the strength of the mQTL association was positively associated with heritability (Fig. 2c, top). There was no such association across ESS probes (Fig. 2c, middle). If the superordinate heritability associated with ESS results from the genetic identity of MZ twins, the mean heritability of ESS probes with mQTL should be higher than that of those without mQTL. This was not the case (Fig. 2c, middle and bottom). This analysis, using mQTL data from *the same samples* in which we identified ESS, provides strong evidence that ESS is not simply a consequence of the isogenicity of MZ twins.

### Testing a model for ESS

During MZ twinning, if de novo DNA methylation at a particular locus occurs prior to embryo cleavage, both twins will inherit the same epigenotype at the locus simply because of developmental timing, rather than as a consequence of their genetic identity [14]. This provides a potential explanation for ESS (Fig. 2d). If correct, methylation at ESS loci must be established in the cleavage-stage embryo. If the epigenetic state is maintained during subsequent cellular differentiation, these loci should show systemic interindividual variation in DNA methylation.

**Fig. 3:**
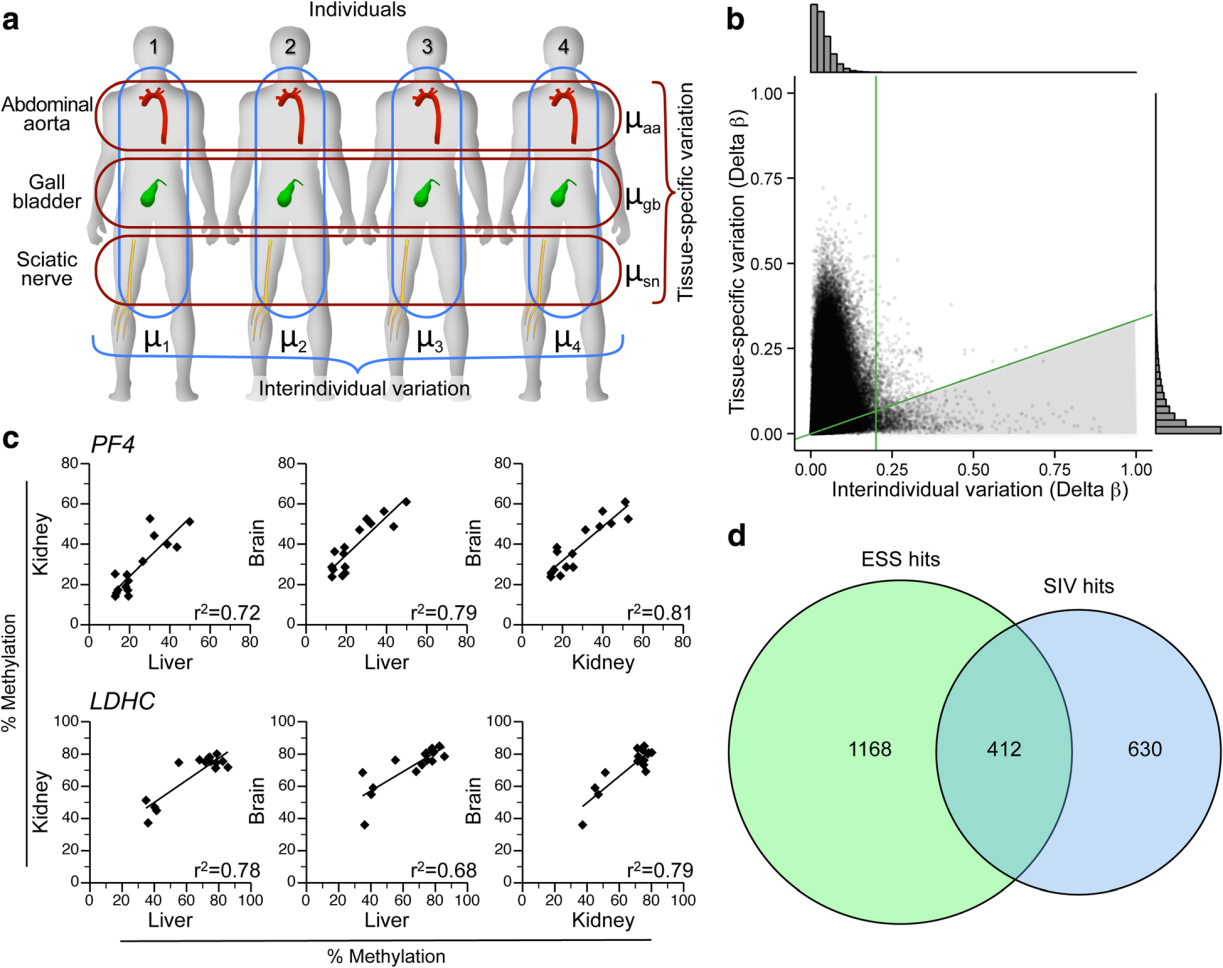
Epigenetically supersimilar (ESS) probes are enriched for systemic interindividual variation (SIV). **a** Analytical strategy applied to data of Lokk et al. [20] on abdominal aorta, gall bladder, and sciatic nerve from each of four individuals. Interindividual and tissue-specific variation were quantified as the range of the individuals’ mean beta values (μ_1_, μ_2_, μ_3_, μ_4_) and the tissues’ mean beta values (μ_aa_, μ_gb_, μ_sn_), respectively. **b** Tissue-specific vs. interindividual variation for 344,151 probes. Histograms (*top* and *side*) indicate the density distribution. *Green lines* illustrate cutoffs used to identify 1042 SIV probes (*shaded region*, interindividual > 0.2 and tissue-specific < 1/3 of interindividual variation). **c** Examples of bisulfite pyrosequencing data confirming systemic interindividual variation in selected loci: *PF4* and *LDHC*. **d** The 1580 probes with evidence of ESS are 6.3-fold enriched for SIV (*P* < 10^–10^, chi-squared test)

To test this, we selected 13 ESS regions and assessed systemic interindividual variation (SIV) by bisulfite pyrosequencing in liver, kidney, and brain of cadaver tissues [13]. Methylation tended to be correlated in these tissues derived from the different embryonic germ layers (Fig. 2e; Additional file 1: Figure S2). Overall, 9 (69%) of the 13 loci showed evidence of SIV (Additional file 2: Table S2). For a broader evaluation, we analyzed a previously published data set from Lokk et al. [20], who profiled multiple tissues from each of several individuals using the HM450 platform. From each of four individuals, we considered data for all SNP-free and high-quality HM450 probes for tissues representing the three embryonic germ layer lineages: gall bladder (endodermal), abdominal aorta (mesodermal), and sciatic nerve (ectodermal). Individual- and tissue-specific methylation were estimated as the average across the three tissues and the four individuals, respectively, and variation was quantified as the range of these averages (Fig. 3a). Though most probes showed little of either (Fig. 3b, histograms), tissue-specific was generally greater than interindividual variation (Fig. 3b). To focus on robust SIV we restricted our analysis to probes with interindividual variation that was at least 0.2 delta β and three times greater than tissue-specific variation (Fig. 3b, shaded region). These cutoffs identified 1042 probes with evidence of SIV (Additional file 2: Table S1). Bisulfite pyrosequencing in cadaver tissues (Fig. 3c; Additional file 1: Figure S3) confirmed SIV at 8 (67%) of 12 regions evaluated (Additional file 2: Table S3).

Perfect overlap between ESS and SIV probe sets was not anticipated for two reasons. First, as they survey only four individuals, the Lokk et al. data cannot capture all interindividual variation. Second, epigenetic states established prior to gastrulation may not be maintained in all differentiated lineages (i.e., early embryonic establishment is necessary but not sufficient for SIV). Nonetheless, relative to the 5388 non-ESS probes with interindividual range > 0.4, the 1580 ESS probes were 6.3-fold enriched for SIV (*P* < 10^–10^, chi-squared test; Fig. 3d), supporting our model for the developmental basis of ESS (Fig. 2d).

### ESS and SIV sites share genomic and epigenomic features, and are enriched for MEs

ESS appears to be a marker for individual-specific epigenetic states that are established in the cleavage-stage embryo. Such states could be established under genetic influence, or stochastically; only the latter are consistent with epigenetic metastability [6]. The mQTL data of Grundberg et al. (Fig. 2c) demonstrate that ESS is not generally associated with genetic effects. To test this more generally we evaluated additional data sets in which the HM450 platform was used to assess mQTL in at least 100 individuals [21]. Volkov et al. [22] profiled SNPs and DNA methylation in adipose tissue of 119 men and identified 15,208 CpG sites with significant *cis*-mQTL. Shi et al. [23] assessed mQTL in histologically normal lung tissue from 210 individuals and reported estimates of the proportion of methylation variance explained by neighboring SNPs (which we refer to as *β*_*SNP*_). We considered probes with *β*_*SNP*_ > 0.33 as exhibiting substantial mQTL. Of the 34,304 probes Shi et al. identified with statistically significant *cis*-mQTL, only 4306 (12.6%) showed substantial mQTL (Additional file 1: Figure S4). Although both the Grundberg et al. [9] and Volkov et al. [22] data were based on adipose tissue, less than half of the mQTL probes identified by either were identified in both (Additional file 1: Figure S5). Conversely, most of the Shi et al. substantial mQTL probes were also identified by the other two studies (Additional file 1: Figure S5). Moreover, > 80% of the probes Shi et al. reported as substantial mQTL in lung also exhibited significant mQTL in independent studies of breast and kidney [23]. For these reasons, we focus our subsequent analyses on the Shi et al. substantial mQTL probe set. (Nonetheless, we have included data on all three mQTL lists in our annotation of ESS and SIV probes in Additional file 2: Table S1.)

**Fig. 4:**
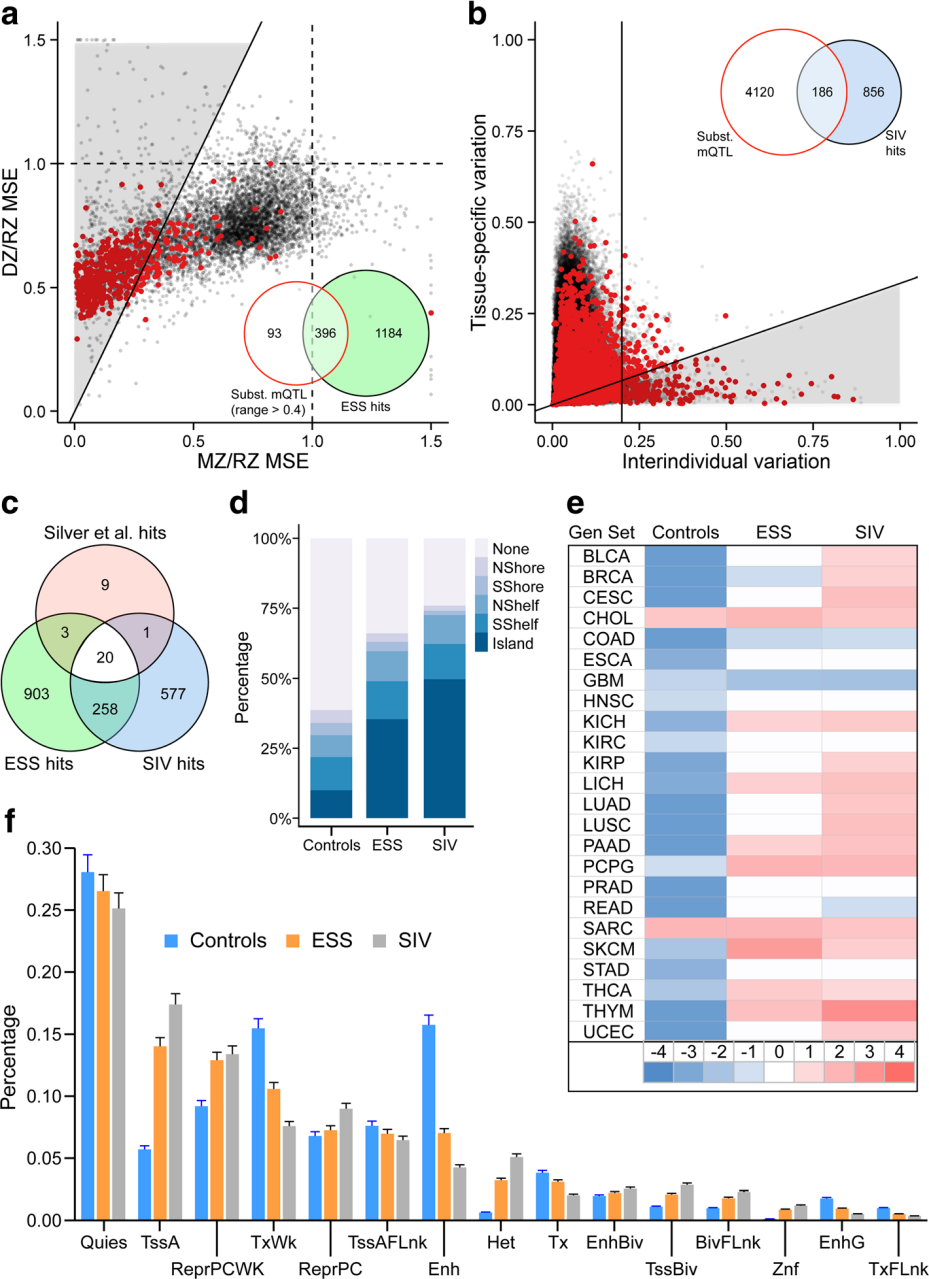
Regions of epigenetic supersimilarity (ESS) and systemic interindividual variation (SIV) share genomic and epigenomic features. **a** Normalized DZ MSE vs. MZ MSE for the 6968 probes with range > 0.4, of which 489 (*red*) show substantial mQTL. *Inset*: ESS probes are 15-fold enriched for substantial mQTL (*P* < 10^–10^, chi-squared test). **b** Tissue-specific vs. interindividual variation at 344,151 probes, of which 2702 (*red*) are substantial mQTL. *Inset*: SIV probes are 24-fold enriched for substantial mQTL (*P* < 10^–10^, chi-squared test). **c** After filtering out substantial mQTL, ESS and SIV hits overlap more than two-thirds of probes at previously identified MEs [13]. **d** Relative to all probes in the top 10% of interindividual variance, ESS and SIV probe sets are enriched for CpG islands (both comparisons *P* < 10^–10^, chi-squared test). **e** Gene set enrichment analysis shows that both ESS and SIV probes are enriched for genes expressed in cancer (*P* = 4.7 × 10^–8^ and 4.8 × 10^–9^, respectively). Each row represents a different type of cancer in The Cancer Genome Atlas [24] (key to abbreviations in Additional file 2: Table S5). **f** Association of probe sets with epigenomic feature annotations derived from 111 reference epigenomes [25]. ESS and SIV probes are enriched for active promoters (TssA) and underrepresented at enhancers (Enh) (all four comparisons *P* < 10^–10^)

**Fig. 5:**
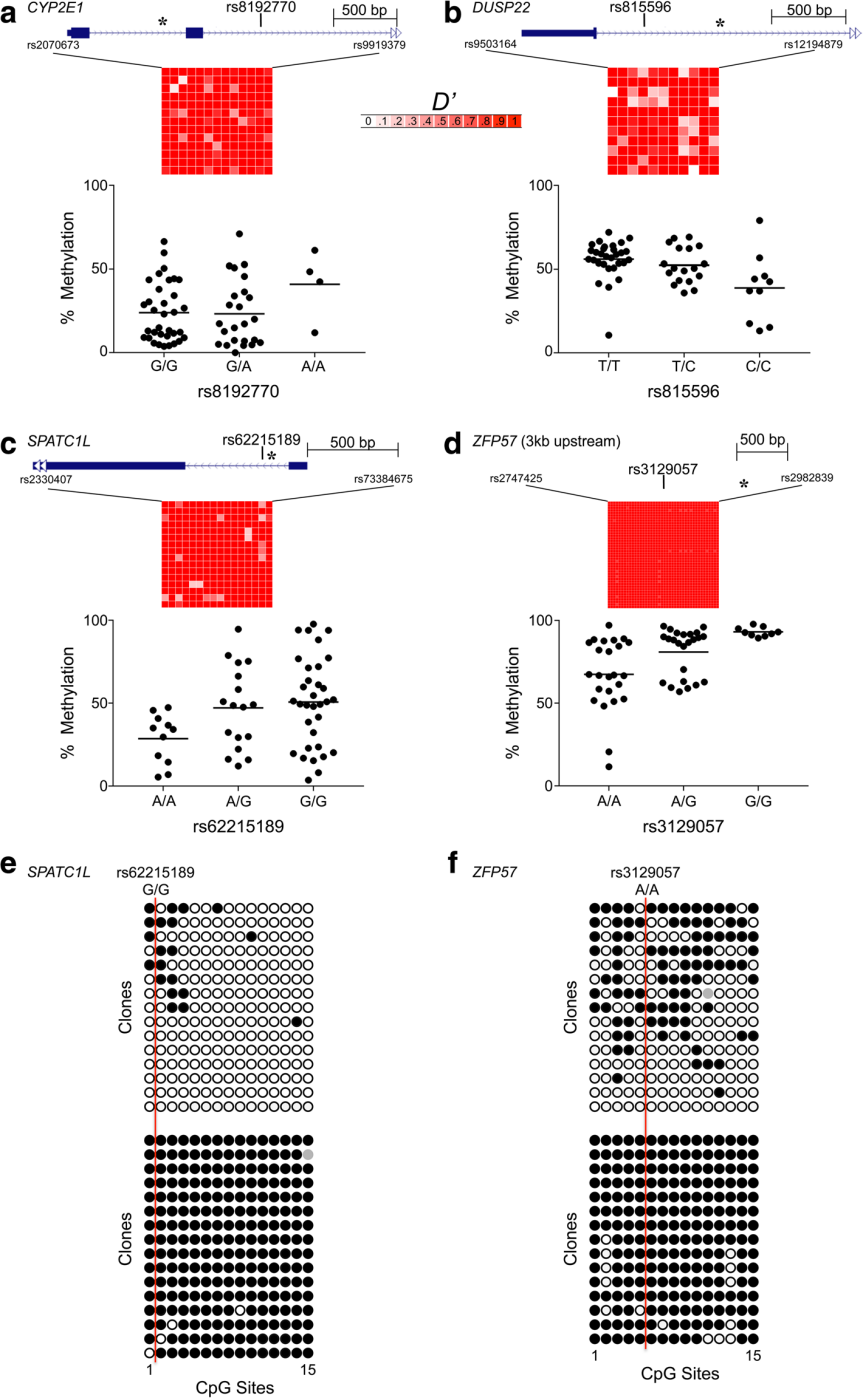
Interactions between DNA methylation and local sequence context at some top ESS regions. **a**–**d** Average methylation vs. SNP genotype at ESS regions within *CYP2E1*, *DUSP22*, *SPATC1L*, and *ZFP57*. In each panel, gene diagram (*top*) shows location of ESS region where methylation analysis was performed (*asterisk*) relative to that of a SNP that was genotyped in 64 Gambian children. Grid summarizes normalized linkage disequilibrium (*D’*) across these ~3-kb regions in a Gambian population in Western Gambia (GWD, 1000 Genomes Project [80]). With the exception of G/G individuals at rs3129057 (*ZFP57*), there is substantial interindividual variation in average methylation within each genotype class. At *CYP2E1* (**a**), average methylation is not associated with SNP genotype (*P* = 0.31). At *DUSP22*, *SPATC1L*, and *ZFP57* (**b**–**d**) average methylation is associated with genotype (*P* = 0.002, 0.02, and 0.0001, respectively). At these same loci, interindividual variance differs between the two homozygous genotypes; i.e., C/C vs. T/T at *DUSP22* (*P* = 0.02), G/G vs. A/A at *SPATC1L* (*P* = 0.04), and G/G vs. A/A at *ZFP57* (*P* = 1.9 × 10^–6^). **e**, **f** Clonal bisulfite sequencing data at two homozygous individuals at each of *SPATC1L* and *ZFP57*, respectively, confirm dramatic interindividual variation in DNA methylation in the absence of local sequence variation. *Black*, *empty*, and *gray circles* represent methylated, unmethylated, and indeterminate CpG sites, respectively. *Vertical red line* indicates the position of the SNP

Relative to the probe sets from which they were drawn, those with evidence of either ESS (Fig. 4a) or SIV (Fig. 4b) were enriched for substantial mQTL (15- and 24-fold, respectively, both *P* < 10^–10^, chi-squared test). Substantial mQTL affected 25.1% of ESS and 17.9% of SIV probes (Additional file 2: Table S1). We consider ESS probes without evidence of substantial mQTL to be candidate MEs. Likewise, since our SIV analysis is analogous to previous ME screens [13, 14], SIV probes without evidence of substantial mQTL are also candidate MEs. Indeed, most of the HM450 probes identified as MEs in a previous screen that employed genome-wide bisulfite sequencing [13] overlap with mQTL-filtered ESS or SIV hits (Fig. 4c). After excluding those with substantial mQTL, ESS probes remained 5.6-fold enriched for SIV (*P* < 10^–10^; Fig. 4c), indicating that the common epigenetic behavior of these probes sets is not due to genetic effects. Importantly, most ESS probes do not show substantial mQTL (Fig. 4a), further evidence that ESS is not simply a consequence of the genetic identity of MZ twins. To directly assess the influence of local sequence on interindividual variation at ESS loci we validated several top hits, performing genotyping and methylation analysis by pyrosequencing in peripheral blood DNA of 64 Gambian children [13]. Each genotyping assay targeted a nearby common SNP with minor allele frequency > 25%. Two regions negative for mQTL (*CYP2E1* and *DUSP22*) and two with some mQTL-positive probes (*SPATC1L* and *ZFP57*) showed substantial interindividual variation even among individuals of the same genotype (Fig. 5a–d). These regions show strong linkage disequilibrium, indicating that SNP genotype is generally an indicator of haplotype. Notably, the SNP we genotyped at *ZFP57*, rs3129057, was recently reported to be the strongest index SNP in phase with haplotype-dependent allele-specific methylation (Hap-ASM) in the region [17]. Significant mQTL was detected for ESS CpGs at *DUSP22*, *SPATC1L*, and *ZFP57* (Fig. 5b–d). At these same loci, however, interindividual variance of methylation was associated with haplotype, providing the novel insight that the local sequence context can influence epigenetic metastability. The pyrosequencing results were further validated by clonal bisulfite sequencing for selected individuals at *SPATC1L* and *ZFP57* (Fig. 5e and f), confirming that even in regions of substantial mQTL, individuals with the same local sequence context can exhibit dramatic interindividual variation in DNA methylation.

Relative to negative control probes with interindividual variation comparable to ESS probes but no evidence of ESS or SIV (Additional file 1: Figure S6 and Additional file 2: Table S4), ESS and SIV probes were 3.6- and 5.0-fold enriched for CpG islands, respectively (Fig. 4d; *P* < 10^–10^ for both comparisons). Likewise, ESS and SIV probes were 3.3- and 2.4-fold enriched in subtelomeric regions (<2 Mb from chromosome ends; Additional file 1: Figure S7a; *P* < 10^–10^ for both comparisons). Since subtelomeric regions are rich in genetic variation, we tested whether the subtelomeric enrichment might be due to mQTL. However, similar enrichments were found in the ESS and SIV probe subsets not associated with substantial mQTL (Additional file 1: Figure S7b). The ESS and SIV gene lists each included six genomically imprinted genes, no different from what is expected by chance; imprinted loci among these two classes are *ANO1*, *GNAS*, *GRB10*, *NAP1L5*, *NLRP*, and *VTRNA2-1* (ESS) and *DLGAP2*, *KCNQ1OT1*, *NAP1L5*, *NLRP2*, and *VTRNA2-1* (SIV) (http://www.geneimprint.com/). Gene set enrichment analysis (GSEA) using data from The Cancer Genome Atlas [24] showed that, relative to negative controls, both ESS and SIV probes are more likely to be annotated to genes expressed in a wide range of tumors (Fig. 4e). Across 111 reference epigenomes encompassing a wide range of cell lines and primary tissues [25], both probe sets were enriched for active promoters and depleted for enhancers (Fig. 4f). ESS and SIV CpGs were identified independently but exhibit highly overlapping genomic and epigenomic features, indicating that they share similar fundamental biological properties.

### Periconceptional environment affects establishment of methylation at ESS and SIV CpGs

**Fig. 6:**
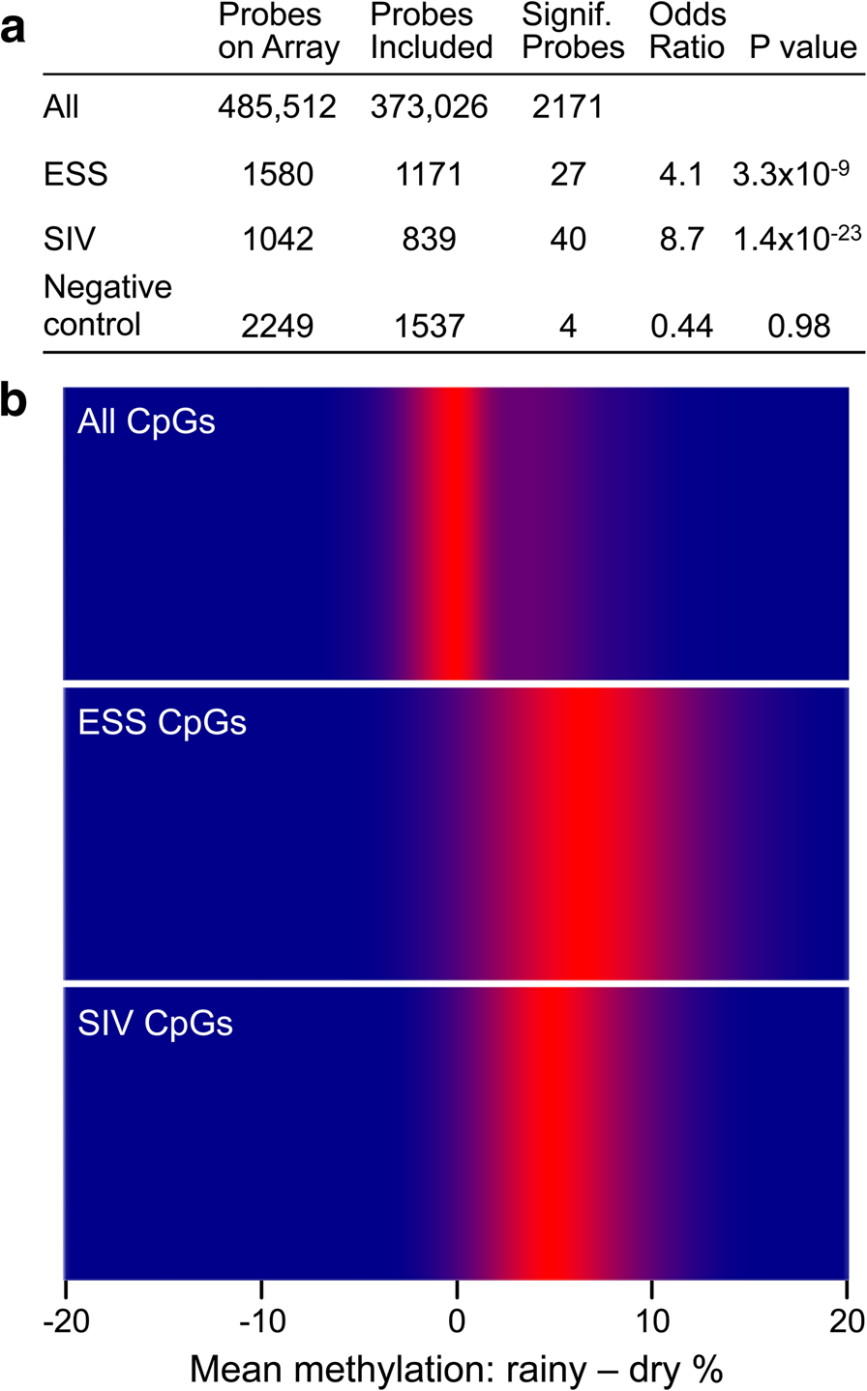
Sites of epigenetic supersimilarity (*ESS*) and systemic interindividual variation (*SIV*) are enriched for effects of periconceptional environment on DNA methylation. **a** Relative to all probes on the HM450 array, mQTL-filtered ESS and SIV probes (but not negative control probes) are highly enriched for significant (FDR < 10%) associations with season of conception in rural Gambia. **b** Heat map of average effect of season of conception at loci that show a significant seasonal difference in methylation (FDR < 10%). At both ESS and SIV probes, as in previous studies of MEs in independent cohorts [13, 14], children conceived in the rainy season have higher methylation

Mouse [10, 11, 26] and human studies [13, 14, 27, 28] have shown that establishment of DNA methylation at MEs is sensitive to periconceptional environment. Previous studies tested this using a “natural experiment” exploiting seasonal variation in maternal nutritional status in The Gambia [12]. Here, we analyzed an independent set of 128 blood samples collected from 2-year-old Gambian participants in the Early Nutrition and Immune Development (ENID) trial [29] who were conceived at the peak of either the rainy or the dry season [30]. Based on the notion that MEs are largely free of genetic influence, we set out to test whether ESS and SIV probes without substantial mQTL show season-of-conception effects. To our surprise, we found comparable and highly significant enrichments for season-of-conception effects in ESS and SIV probe sets regardless of substantial mQTL (Additional file 2: Tables S17 and S18). We therefore examined the unfiltered probe sets and found that both ESS and SIV probes, but not negative control probes, were significantly enriched for season-of-conception effects (*P* = 3.3 × 10^–9^ and 1.4 × 10^–23^, respectively; Fig. 6a). Consistent with previous studies of candidate MEs in independent cohorts [12–14], being conceived during the rainy season generally resulted in higher levels of DNA methylation at both ESS and SIV loci (Fig. 6b). These findings further support the conjecture that, regardless of mQTL, many ESS and SIV probes are MEs. This season of conception effect also provides independent support for our model (Fig. 2d) that ESS arises due to early embryonic establishment of epigenotype.

### Prospective associations between DNA methylation in blood and later cancer

Although the probe signature of ESS was identified from a study of adult twins, it appears to be a consequence of methylation establishment in the early embryo and hence must be stable from embryonic development to adulthood. Since ESS is associated with genes expressed in tumors (Fig. 4e), we asked whether interindividual variation in DNA methylation at ESS loci predicts risk of later cancer in adults. To test this, we examined data from the Melbourne Collaborative Cohort Study (MCCS), which enrolled 41,514 healthy adult volunteers between 1990 and 1994 [31]. Peripheral blood samples and information on health-related behaviors were collected at enrollment, and incident cases of cancer were ascertained prospectively by linkage to the Victorian Cancer Registry, which receives mandatory notification of all new cancer cases in Victoria, Australia. The systemic nature of interindividual variation at ESS probes enabled us to use DNA methylation in peripheral blood as an indicator of methylation in various tissues. A control was matched to each incident case on sex, country of birth, and age at enrollment, using density sampling. Using the Illumina HM450 platform, DNA methylation at baseline was assessed on 3464 case-control pairs overall in studies of seven types of cancer [32] (Additional file 2: Table S6); average time from sample collection to diagnosis was 9.2 ± 4.9 years (mean ± sd).

**Fig. 7:**
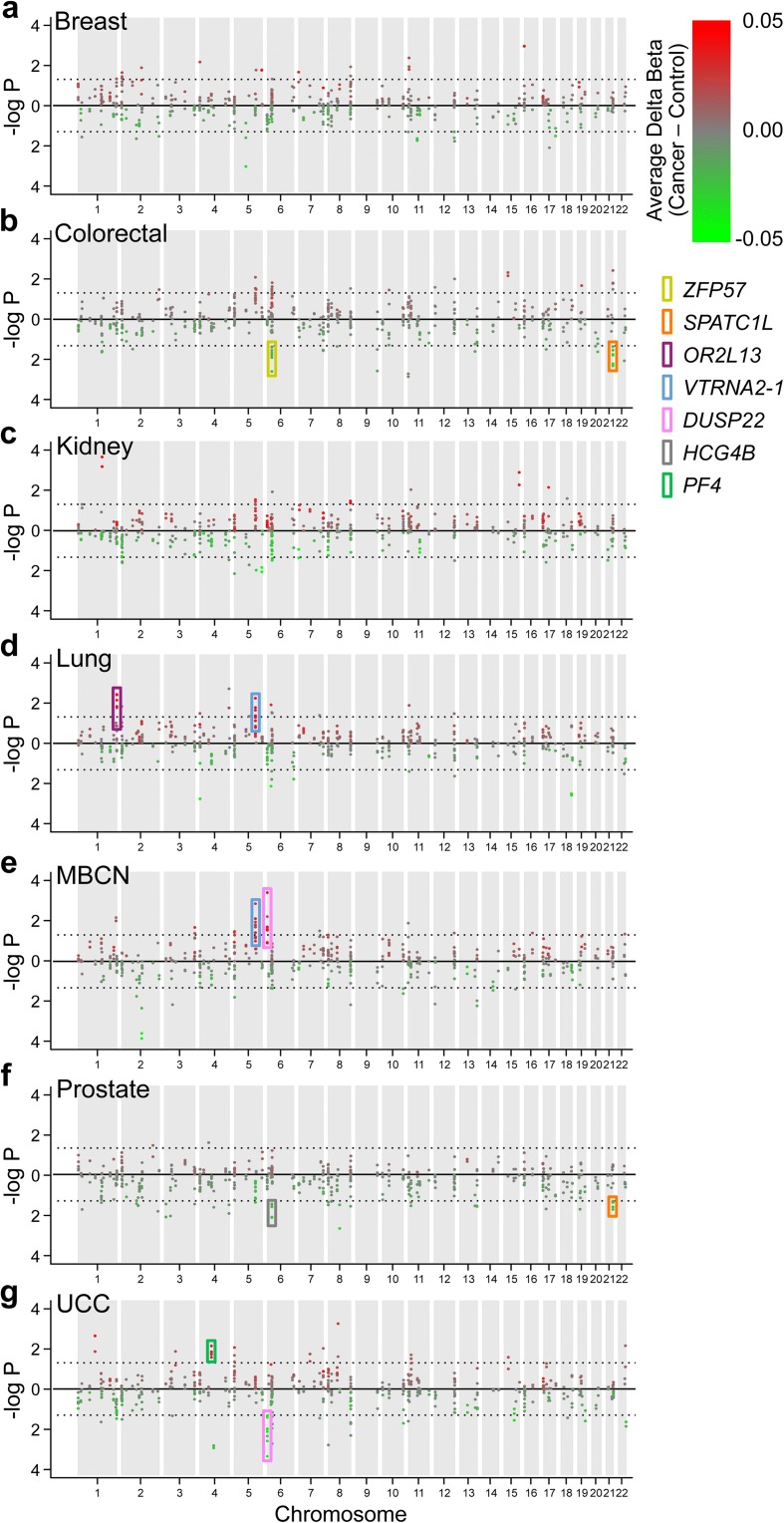
At clusters of probes showing epigenetic supersimilarity (ESS), peripheral blood methylation at baseline is associated with risk of later cancer. Manhattan plots illustrating results of conditional logistic regression analyses of the association between baseline probe-specific methylation (HM450) and risk of later **a** breast cancer, **b** colorectal cancer, **c** kidney cancer, **d** lung cancer, **e** mature B-cell neoplasm, **f** prostate cancer, and **g** urothelial cell carcinoma. Only probes within clusters of ≥ 2 probes are shown. Probes plotted with positive values (*red*) have positive coefficients (i.e., more methylation in cases than controls) and probes plotted with negative values (*green*) have negative coefficients (delta beta value scale indicated). The *dotted lines* indicate *P* = 0.05. Among the ten most CpG-rich ESS clusters, colored boxes indicate seven at which methylation is significantly associated with later cancer: *ZFP57* (colorectal cancer, *P* = 0.008), *SPATC1L* (colorectal cancer, *P* = 0.009, and prostate cancer, *P* = 0.01), *OR2L13* (lung cancer, *P* = 0.010), *VTRNA2-1* (lung cancer, *P* = 0.025, and MBCN, *P* = 0.009), *DUSP22* (MBCN, *P* = 0.001, and UCC, *P* = 0.001), *HCG4B* (prostate cancer, *P* = 0.007), and *PF4* (UCC, *P* = 0.013)

Regardless of potential genetic influences, our data indicate that interindividual epigenetic variation at ESS probes occurs systemically and is stable over time. We therefore evaluated ESS probes without regard to mQTL. Combined effects across multiple CpGs (i.e., differentially methylated regions) are more likely to demonstrate long-term stability and affect gene expression [33]. Hence, rather than analyze individual probes, we focused on the 198 clusters of multiple ESS probes separated by no more than 500 bp (523 CpGs total; Additional file 2: Table S7). Analysis of expression [34] and methylation data [9] from Grundberg et al. showed that at many ESS clusters, average methylation in adipose tissue is associated with gene expression, not only in adipose tissue but also in skin and lymphoblastoid cell lines (Additional file 1: Figure S8 and Additional file 2: Table S8). These results provide evidence that methylation at ESS loci in one tissue yields information about epigenetic regulation in additional tissues. To test for probe-specific associations between peripheral blood DNA methylation at baseline and risk of specific cancer diagnosis we performed conditional logistic regression, adjusting for estimated leukocyte composition (using the Houseman algorithm [35]) and other covariates. Statistical significance of associations at the cluster level were then evaluated by permutation testing (tabulated results in Additional file 2: Table S11). Relative to negative control clusters (Additional file 2: Table S12), the 198 ESS clusters were four-fold enriched for associations with later cancer (*P* = 1.5 × 10^–5^). To minimize multiple testing, we focused on the ten ESS clusters with the largest number of CpG sites. Remarkably, at seven of these, peripheral blood DNA methylation at baseline was significantly associated with later cancer (Fig. 7; Additional file 2: Table S13); three (*SPATC1L*, *VTRNA2-1*, and *DUSP22*) were significantly associated with multiple types of cancer. Elevated methylation in a cluster of six CpG sites at *SPATC1L* was associated with reduced risk of colorectal and prostate cancer (Fig. 7b, f), and elevated methylation in a cluster of 12 CpGs at *VTRNA2-1* was associated with higher risk of lung cancer and mature B-cell neoplasm (Fig. 7d, e). Interestingly, elevated methylation in a cluster of eight CpG sites at *DUSP22* was associated with increased risk of mature B-cell neoplasm (Fig. 6e) yet reduced risk of urothelial cell carcinoma (Fig. 7g). The 154 negative control clusters showed few and relatively weak associations with later cancer (Additional file 1: Figure S9). Results are also shown for the 128 ESS clusters that included no probes with substantial mQTL (Additional file 1: Figure S10 and Additional file 2: Table S14).

## Discussion

Because they offer the potential to test the hypothesis that interindividual epigenetic variation (in the absence of genetic variation) determines human phenotype, MZ twins have long been a focus of epigenetic investigation [36–40]. Such studies depend upon the existence of stochastic (i.e., non-genetically mediated) epigenetic differences within pairs of MZ twins. Conversely, herein we have identified a set of human genomic regions at which MZ twins exhibit non-genetically mediated epigenetic *similarity*. Based on the frequent occurrence of SIV in ESS regions, and their epigenetic plasticity to periconceptional environment, we propose that ESS arises due to establishment of DNA methylation prior to embryo cleavage during MZ twinning.

Accordingly, at ESS loci one would expect greater epigenetic similarity in MZ twins that separate later compared to those that separate earlier. This can be tested based on chorionicity; cleavage before day 4 of gestation results in MZ twins each with their own placenta (dichorionic); later cleavage results in a shared placenta (monochorionic). In one of the earliest genome-scale studies of DNA methylation in twins, Kaminsky et al. [38] studied buccal epithelial cells and reported that monochorionic MZ twins exhibit greater epigenetic discordance than dichorionic, contrary to our thesis. A slightly larger study, however, recently assessed genome-scale DNA methylation in blood and came to the exact opposite conclusion [41]. Given that monochorionic twins share hematopoietic stem cells during fetal development [42], blood is not the ideal tissue in which to study epigenetic correlates of chorionicity. Definitive studies in non-blood tissues and focused on ESS regions are needed. Another predicted consequence of ESS is that estimates of methylation heritability from twin studies will be inflated relative to those from family-based designs. Indeed, whereas Grundberg et al. estimated median genome-wide narrow-sense *h*^*2*^ = 0.34 [9], a recent large family-based study (also using the HM450 platform) estimated an average genome-wide *h*^*2*^ = 0.19 [43].

After decades of epigenetic studies in MZ twins, it is remarkable that ESS has not been previously reported. Despite their seemingly unsupportive findings in monochorionic vs. dichorionic twins, Kaminsky et al. proposed that in addition to their genetic identity, “epigenetic similarity at the time of blastocyst splitting may also contribute to the phenotypic similarities in MZ co-twins,” exactly as our findings suggest. The excessive *h*^*2*^ estimates in twin studies of epigenetic heritability have, in fact, been waiting to be discovered. Grundberg et al. obtained but did not comment upon HM450 probe-specific *h*^*2*^ estimates > 1. Likewise, in a more recent study using the HM450 array to assess genome-scale DNA methylation in whole blood of MZ and DZ twins, van Dongen et al. [44] reported 3792 probes for which their heritability model failed to converge. Of the 631 of these “NA” probes among the high-variance set from which our ESS probes were drawn, 365 (58%) are classified as ESS. Hence, two recent large studies of DNA methylation in MZ and DZ twins detected but did not explore these very interesting genomic regions.

Our findings indicate complex relationships among genetic variation, ESS, and epigenetic metastability. To clarify, mQTL assesses pairwise associations between methylation at a specific CpG site and a specific genetic variant [19], while hap-ASM describes allelic biases in methylation that are associated with haplotype [17]. Because of the linkage disequilibrium among neighboring SNPs and the regional correlation of CpG methylation, mQTL (specifically, *cis*-mQTL) provides a means of assessing hap-ASM [17]. Our analyses focused on mQTL because many targeted analyses of hap-ASM [21] show poor overlap with probes on the HM450 platform [17]. Our results show that ESS loci are enriched for mQTL. Although this may seem to suggest that ESS is a consequence of genetic determination, we’ve provided several lines of evidence to the contrary. According to our model (Fig. 2d) mQTL is consistent with ESS, because any epigenetic state (whether under genetic influence or not) that is established prior to embryo cleavage during MZ twinning and thereafter maintained with high fidelity will exhibit ESS. Seminal studies in isogenic mice led to the concept that interindividual variation at MEs is determined stochastically, free of genetic influence [45]. Our characterization of ESS loci (many of which appear to be MEs) suggests the novel concept that establishment of epigenotype at MEs need not be completely free of genetic influence. In particular, mQTL and epigenetic metastability appear to occur at the same loci (Fig. 5) and ESS loci—even those associated with substantial mQTL—are labile to perioconceptional environment (Fig. 6; Additional file 2: Tables S17 and S18). Like nutrition [10–12] and other environmental influences [27, 46], perhaps haplotype (i.e., local sequence context) may be viewed as a determinant of the microenvironment that shifts the probability distribution of stochastic methylation processes during early embryonic development. Building upon this, our validation studies indicate allelic biases in epigenetic metastability. In the clearest example, at *ZFP57* (Fig. 5d), the most common allele in the population showed greater interindividual variation, consistent with the thesis that propensity for stochastic epigenetic variation may be both genetically inherited and evolutionarily advantageous [47].

It may seem surprising that ESS loci include some genomically imprinted genes. Based on their parent-of-origin-specific epigenetic regulation one would expect the mean MSE at imprinted loci to be similar in MZ and DZ twin pairs. Our data at *VTRNA2-1*, however, show this is clearly not the case (Fig. 1a). Known imprinted genes were not significantly enriched among ESS loci, but there is evidence that two more of our top hits (*PAX8* and *DUSP22*) are imprinted in humans, in at least some tissues [48, 49]. Our interpretation is that interindividual variation at imprinted loci may in some cases occur stochastically; for example, the *VTRNA2-1* hypomethylation that is observed in 10–20% of individuals [13, 50, 51] may reflect loss of the maternally inherited methylation mark in the early embryo. At the population level many ESS loci exhibit clusters of three methylation states (Fig. 1 and Additional file 1: Figure S8). This suggests these loci behave as bistable epigenetic switches (i.e., the combination of two alleles yields three preferred average states). This is actually consistent with the bimodal distribution of somatic CpG methylation genome-wide (i.e., methylation at most loci is either close to 0 or close to 100%). In this regard the presence of imprinted loci—paradigmatic bistable epigenetic switches—among ESS loci is not surprising.

Although identified purely on the basis of the methylation MSE ratio of adult DZ to MZ twins, ESS probes are threefold enriched in subtelomeric regions. This makes sense; subtelomeric regions are packed with transposable elements, known to be targets of de novo DNA methylation in the pre-implantation embryo [52]. We found a similar enrichment in our genome-wide screen for MEs [13] but filtered out most of those hits due to proximity to SNPs. Our current results, showing that the subtelomeric enrichment is not associated with mQTL, suggest we were overly conservative. Intriguingly, since epigenetic regulation in subtelomeric regions regulates telomere shortening [52], our Gambian data showing season of conception effects at ESS regions suggest that periconceptional events could influence the process of telomere maintenance, deregulation of which is an almost universal characteristic of aging and cancer.

Because of their early embryonic establishment and systemic interindividual variation, ESS loci are attractive candidate regions for studies of epigenetics and disease. The HM450 array was built upon a platform initially focused on regions aberrantly methylated in cancer, motivating our focus on cancer. Methylation at three clusters (*SPATC1L*, *VTRNA2-1*, and *DUSP22*) showed significant associations with two types of cancer. Little is known about the speriolin-like protein SPATC1L, but elevated methylation at the small noncoding RNA *VTRNA2-1* has previously been reported to predict poor prognosis in acute myeloid leukemia [51] and other types of cancer [13], consistent with the positive association we found between *VTRNA2-1* methylation and lung cancer and mature B-cell neoplasm (Fig. 7d, e). Likewise, rearrangements disrupting the dual specificity phosphatase gene *DUSP22* are associated with T-cell and B-cell lymphoma [53], consistent with our finding of a positive association between methylation at *DUSP22* and mature B-cell neoplasm (Fig. 7e). Methylation at *DUSP22* was also *negatively* associated with risk of urothelial cell carcinoma (Fig. 7g), reminiscent of situations in which the same genetic variant is associated oppositely with risk of different types of cancer [54]. *ZFP57* encodes a master regulator of genomic imprinting and is epigenetically labile to periconceptional nutrition [13]. Our finding that elevated methylation at *ZFP57* is associated with a reduced risk of later colorectal cancer (Fig. 7b) is consistent with data suggesting *ZFP57* is an oncogene [55]. Likewise, PF4 (platelet factor 4) inhibits tumor growth and metastasis by suppressing neovascularization [56], consistent with the positive association we found between *PF4* methylation and later urothelial cell carcinoma (Fig. 7g). Despite detecting significant associations between methylation and later cancer, the effect sizes in most cases were modest. It is likely that effects of some epimutations are limited to specific cancer subtypes. Likewise, these epigenetic variants likely interact with genetic variation and environmental exposures to affect cancer risk. It is possible that some of these associations might reflect mQTL/hap-ASM association with cancer-associated SNPs identified in GWAS studies. Targeted studies in larger cohorts are needed. Nonetheless, our data indicate that individual epigenetic variation at ESS loci has phenotypic consequences: methylation in peripheral blood is associated with risk of specific cancer diagnoses years later. Despite their rarity (less than 1% of the probes on the HM450 array), broader-scale identification of ESS loci throughout the genome may enable epigenetic risk models for cancer prediction, even during early life.

Our findings may offer a partial explanation for missing heritability. Since heritability is defined as the phenotypic variance explained by genetics divided by the total phenotypic variation in a population [16], rather than something heritable per se, what is actually missing is genetic variance [57]. Twin models of estimating heritability rely on the assumption that the greater phenotypic similarity of MZ relative to DZ twin pairs is attributable to their genetic identity. Hence, to the extent that epigenetic variation at ESS loci influences phenotype, estimates of heritability based on twin studies will be inflated. Indeed, twin studies often yield higher heritability estimates than family studies [58, 59]. Further, although heritability does not definitively connote transgenerational inheritance, transmission of sequence-independent epigenetic events across generations could contribute to missing heritability [57]. In this regard, genomically imprinted loci that behave as epialleles (such as *VTRNA2-1*) could potentiate transgenerational inheritance of epigenetic traits.

## Conclusions

Overall, our data show for the first time that, independent of their genetic identity, human MZ twin pairs share an additional level of similarity at the epigenetic level. ESS appears to result from establishment of mitotically heritable epigenetic states prior to embryo cleavage during MZ twinning. Because of ESS, human MZ twins clearly cannot be viewed as the epigenetic equivalent of isogenic inbred mice, which originate from separate zygotes. To the extent that epigenetic variation at ESS loci influences human phenotype, as our data indicate, the existence of ESS establishes a link between early embryonic epigenetic development and adult disease and may call into question heritability estimates based on twin studies.

## Methods

### Identification and characterization of ESS and SIV probes

#### *Analysis of twin*, *SIV*, *and mQTL data sets*

Grundberg et al. used the Illumina HM450 array to assess methylation in adipose tissue from 662 female twins of European-descent, including 97 MZ pairs and 162 DZ pairs. Methylation scores were normalized by quantile normalization. SNP-associated probes were excluded, leaving 344,303 probes [9]. Our analyses focused on the 34,405 probes in the top 10% by variance. To calculate the metrics used, we pooled the MZ and DZ twins into a single population, and calculated probe-specific β-value ranges (max − min) from this population. Individuals in this population were randomly paired to simulate unrelated individuals (RZ). For each probe, we calculated the MSE of MZ, DZ, and RZ pairs from the line of identity (i.e., the mean square difference between twins). For *n* twin pairs, each comprised of twins *A* and *B*:

$$ \mathrm{MSE}=\frac{1}{n}\sum \limits_{i=1}^n{\left({\upbeta}_{iA}-{\upbeta}_{iB}\right)}^2 $$

Criteria for ESS probes were DZ/MZ > 2 and overall interindividual β-value range (max − min) > 0.4; additionally, 14 probes with MZ/RZ MSE > 0.5 were excluded. Probe-specific *h*^*2*^ estimates from [9] were kindly provided by Elin Grundberg. Lokk et al. [20] used the Illumina HM450 array to assess methylation in 17 tissues from four autopsied individuals. We analyzed the methylation data for three tissues representing the three germ layers: gall bladder (endodermal), abdominal aorta (mesodermal), and sciatic nerve (ectodermal). Starting with the 344,303 high-quality probes that were the basis of the Grundberg et al. analysis, we excluded any probes with missing values in any of the 12 samples (four individuals, three tissues), leaving 344,151 probes. Interindividual variation was calculated by taking the mean β value across each individual’s three tissues, then calculating the range of these means across all four individuals. Tissue-specific variation was calculated by taking the mean beta value of each tissue over all individuals, then calculating the range of these means across all three tissues. Negative control probes (Additional file 2: Table S4) were selected by maintaining the criterion of interindividual range > 0.4 in the Grundberg et al. data set, but requiring MZ/RZ MSE > 0.5 and (in the Lokk data set) requiring tissue-specific variation to be at twice interindividual variation (Additional file 1: Figure S6). Figures were made in R 3.3.1 using ggplot2 [60]. For our analysis of the Shi et al. mQTL data [23], senior author Maria Landi kindly shared with us their estimates of the proportion of methylation variance explained by neighboring SNPs.

#### Validation studies

Quantitative analysis of selected candidate MEs was performed by bisulfite pyrosequencing [61] across endodermal (liver), mesodermal (kidney), and ectodermal (brain) tissue in 17 Asian cadavers [13]. Prior to use, all pyrosequencing assays were validated for linearity and sensitivity using human genomic methylation standards [12, 13] (Additional file 2: Table S15). To assess SIV, for each pyrosequencing assay methylation was averaged across multiple CpG sites for each sample, and inter-tissue correlation coefficients were calculated across the 17 cadavers (kidney vs. liver, brain vs. liver, and brain vs. kidney). Regions yielding an inter-tissue correlation of R^2^ > 0.50 (R > 0.71) were considered positive for SIV [12]. Pyrosequencing was also used to perform SNP genotyping at specific loci [13, 14]. Associations between SNP genotype and average methylation were evaluated by linear regression (SAS), and effects of genotype on variance were evaluated by Bartlett’s test (implemented in R). Clonal bisulfite sequencing was performed as previously described [62], using primers listed in Additional file 2: Table S15.

#### Gene set enrichment analysis

For each of the probe sets analyzed (e.g., ESS, SIV, and negative controls), associated gene sets were determined based on the HM450 probe annotations. For 24 cancer types profiled by The Cancer Genome Atlas [24], we downloaded the RNA-Seq gene expression profiles using the firebrowse.org portal [63], selected genes with maximum FPKM across all samples exceeding 1, then generated rank file for gene set enrichment analysis (GSEA) [64] as previously described [65] by comparing the tumor samples and the adjacent normal samples. Next, GSEA was run for each cancer type rank file and each CpG-associated gene set; significance was considered for q-value < 0.25. For visualization purposes, we represented the normalized enrichment score (NES) for each significant enrichment; by convention, NES for enrichments with Q ≥ 0.25 were considered 0.

#### Epigenomic distribution of CpG probes

For each of the probe sets analyzed (e.g., ESS, SIV, and negative controls), genomic coordinates on the human genome build UCSC hg19 were determined based on the HM450 probes definition. We considered fifteen-state genome-wide epigenomic partitions for 127 distinct epigenomes as defined by the NIH Epigenomic Roadmap Consortium [25], based on a collection of uniformly collected histone modification ChIP-Seq profiles and using the ChromHMM algorithm [66]. Using the BEDTOOLS software, we determined the relative distribution of each CpG probe set over the 15 epigenomic states for each distinct epigenome.

#### Evaluating the relationship between DNA methylation and gene expression at ESS clusters

As described above, we used HM450 methylation data for subcutaneous adipose tissue from MZ and DZ twins [9]. Gene expression data in skin, adipose tissue, and lymphoblastoid cell lines from the same set of twins was downloaded from ArrayExpress (accession E-TABM-1140) [34, 67]. DNA methylation (β values) were first averaged across probes within each ESS cluster. Correlation between cluster-level DNA methylation and associated gene expression was evaluated using the Spearman (rank) correlation coefficient, as implemented in the Python scientific libraries.

### Season of conception analyses

#### Sample selection and preparation

The data were collected as part of a study in The Gambia (in sub-Saharan West Africa) identifying biomarkers and understanding mechanisms for the relationship between aflatoxin exposure and child stunting; 251 blood samples (3 ml) were collected from children aged 2 years as part of the Early Nutrition and Immune Development (ENID) Trial [29].

#### Bisulfite conversion and DNA methylation assay

DNA was extracted from white blood cells following a previously described protocol [12]. An additional six samples were included as technical replicates. Genome-scale methylation profiles were obtained using HM450 Infinium methylation bead arrays (Illumina, San Diego, USA). DNA samples (500 ng) were bisulfite-modified using the EZ DNA Methylation kit (Zymo Research, D5001) following manufacturer’s instructions for the HM450 array. Modified DNA was stored at −20 °C until used. Amplification, labeling, hybridization and scanning were performed as previously described [13]. These data sets are publicly available [30].

#### Methylation data quality control and normalization

Methylation data pre-processing was performed using the R/Bioconductor *minfi* package [68], along with other functions and bespoke R scripts as appropriate (R version 3.2.3; Bioconductor version 3.2). Briefly, data for 485,512 HM450 probes measured in 257 samples were imported from raw IDAT files. Analysis of internal HM450 bisulfite conversion control probes revealed one sample with poor bisulfite conversion efficiency, which was excluded. Functional normalization [69] was used to reduce unwanted technical variation using control probes on the array. Seven samples with discordant sex were removed following sex prediction based on median values of measurement on X and Y chromosomes using the *minfi* addSex() function. Using a probe detection *P* value threshold of 0.01, five samples failing in > 1% of probes were removed, along with 32,488 probes failing in one or more samples. All technical replicates showed beta-value Pearson correlations > 0.994 and visual inspection of replicate correlation scatterplots revealed no anomalies. Following removal of technical replicates and X and Y chromosome probes, methylation data for 442,869 probes measured in 239 individuals remained. Correction for differences in HM450 beta-value distributions due to type-I and type-II probes on the array was conducted using the Beta Mixture Quantile Dilation (BMIQ) method [70]. Finally, 28,509 cross-reactive probes [71] and 41,334 probes within 10 bp of common (minor allele frequency > 1%) African SNPs identified using the R *Illumina450ProbeVariants.db* were removed. After all quality control and filtering, 373,026 probes remained.

#### Identification of CpGs associated with Gambian season of conception

Statistical analysis was performed to identify HM450 probes associated with Gambian season of conception, described hereafter as “season of conception differentially methylated probes” (SoC-DMPs). This analysis was restricted to 128 individuals conceived at the peak of either the Gambian dry (February–April) or rainy (July–September) seasons (based on date of birth). These seasonal windows have been used in previous studies investigating seasonal effects on DNA methylation [12, 13]. Robust linear regression using the R *rlm* function was used to model the association between SoC and DNA methylation (measured as HM450 beta-values), in order to account for potential heteroscedasticity and influential outliers [72, 73]. The regression model included infant sex, and the first three principal components identified in an unsupervised principal components analysis of genome-wide methylation (Additional file 2: Table S16 and Additional file 1: Figure S11). The model was additionally adjusted for the effects of cell heterogeneity using an established method that uses methylation data to estimate the relative proportions of six white blood cell types [35]. Additional analyses were performed i) without cell composition adjustment to assess sensitivity to cell composition effects; and ii) with the inclusion of one further principal component (PC8) which was associated with SoC, which would be expected to dilute the SoC effect (hence providing a more conservative estimate of SoC-associated ME enrichment). A correction for multiple testing was applied by controlling the false discovery rate (FDR).

#### Enrichment analysis

Probes with an FDR < 10% were identified as SoC-DMPs. Different sets of HM450 probes were tested for SoC-DMP enrichment (FDR < 10%) using Fisher’s exact test, against a background of all 373,026 probes passing quality control and filtering steps. Enrichment results for the main analysis are presented in Fig. 6a. Results for additional enrichment tests performed without adjustment for cell composition and with the inclusion of one further principal component (see previous section) are presented in Additional file 2: Tables S17 and S18.

### Identification of ESS clusters associated with cancer risk

#### *Sample collection*, *data generation*, *and quality control*

Methylation data were available for participants in one of seven case-control studies of breast, colorectal, kidney, lung, mature B-cell malignancies, prostate or urothelial cancer [74–76] nested within the Melbourne Collaborative Cohort Study [31]. DNA was extracted from samples of peripheral blood mononuclear cells (PBMC), buffy coats, or dried blood spots (DBS) stored on Guthrie card diagnostic cellulose filter paper. Samples were collected at recruitment to the cohort (baseline) or at follow-up approximately 10 years later. Cases and controls were individually matched on age (they had to be free of cancer at an age within one year of the age at diagnosis of the corresponding case), sex, ethnicity, and blood DNA source (DBS, PBMC, or buffy coat). For all but the colorectal cancer study, controls were matched to cases on year of birth. For the lung cancer study, controls were matched on smoking status at the time of blood collection. Case-control pairs were placed at random positions on the same chip of the assay to minimize batch effects.

DNA was extracted from mononuclear cells using QIAamp mini spin columns (Qiagen, Hilden, Germany). Dried blood spot DNA was extracted as previously described [77]. Briefly, 20 blood spots of 3.2 mm diameter were punched from the Guthrie card and lysed in phosphate-buffered saline using TissueLyser (Qiagen). The resulting supernatant was processed using Qiagen mini spin columns according to the manufacturer’s protocol. The quality and quantity of DNA were assessed using the Quant-iT™ Picogreen® dsDNA assay measured on the Qubit® Fluorometer (Life Technologies, Grand Island, NY), with a minimum of 0.3 μg DNA considered acceptable for methylation analysis.

Bisulfite conversion was performed using the Zymo Gold single tube kit (EZ DNA Methylation-Gold kit, Zymo Research, Irvine, CA, USA) according to the manufacturer’s instructions. Post-conversion quality control was performed using SYBR Green-based quantitative PCR, an in-house assay designed to determine the success of bisulfite conversion by comparing amplification of the test sample with positive and negative controls. All samples were processed in the same laboratory on 96-well plates, each using eight HM450 BeadChips to assay batches of 12 samples.

The methylation data were background corrected and normalized based on internal control probes using the manufacturer’s background correction, using the R library minfi [68]. We also applied subset-quantile within-array normalization (SWAN) [78] to correct for technical discrepancies between type I and type II probes on the assay. A β-value (interpreted as percentage methylation) was calculated for each CpG site using minfi. Methylation measures with a detection *P* value higher than 0.01 were considered missing. Samples with more than 5% missing values were excluded; then, CpGs that were missing for more than 20% of samples were excluded. β-values were transformed into M-values for analysis: M = log2(β/(1 − β)).

#### Logistic regression and permutation analyses

For each CpG probe set, we first determined their clustering structure by considering all CpGs within 500 bp of each other; groups of at least two such CpGs were considered clusters. ESS, SIV, and negative control cluster annotations are provided in Additional file 2: Tables S7, S8, and S9. For each of the seven case-control cohorts described above, normalized DNA methylation data at the CpG probe level were obtained. These data sets are publicly available [32]. Since methylation was measured in peripheral blood DNA, cell type composition estimates using established methods [35] were also included for each sample (specifically, proportions of B cells, granulocytes, monocytes, NK, CD4 T cells, and CD8 T cells). Clinical data variables indicating body mass index (BMI), alcohol consumption, and smoking status were included for each subject. Many ESS probes showed highly non-normal methylation distributions within each cohort. To avoid incorrect assumptions about the data distribution, the M-values for each probe were rank-normalized in ascending order across all samples using the R statistical system. Using conditional logistic regression as implemented in the *R survival* package, we determined for each probe the significance of the association between methylation rank and cancer status, in a model including both cell type proportion and the clinical variables described above. For the purposes of permutation testing (see below), associations were considered statistically significant at *P* < 0.05.

These probe-specific *P* values were then utilized to evaluate the statistical significance of associations at the cluster level. We focused our analysis on the top ten clusters by total number of CpGs. We assessed the significance of two event types. For each cluster (*C*), cancer type (*T*), and random assignment of the case-control status for each matched pair (*S*_*rnd*_), we denoted the number of significant probes at *P* < 0.05 with concordant coefficients as determined by conditional logistic regression as *N(C*,*T*,*S*_*rnd*_*)*. We denote the actual case-control status from the MCCS cohort as *N(C*,*T*,*S*_*obs*_*)*. We likewise denote the minimum *P* value obtained across all the probes in a cluster, for the randomly assigned and actual case-control pairing as *P*_*min*_*(C*,*T*,*S*_*rnd*_*)* and *P*_*min*_*(C*,*T*,*S*_*obs*_*)*. We defined the event:

i) *N(C*,*T*,*S*_*rnd*_*)* ≥ *N(C*,*T*,*S*_*obs*_*)* and *P*_*min*_*(C*,*T*,*S*_*rnd*_*)* ≤ *P*_*min*_*(C*,*T*,*S*_*obs*_*)*
  Next, for each random assignment *S*_*rnd*_ we defined the event *Recurrence(C*,*S*_*rnd*_*)* as the number of cancer types for which a cluster C contains at least two significant probes (*P* < 0.05) with concordant coefficients. The corresponding value for the actual case-control status is *Recurrence(C*,*S*_*obs*_*)*. We defined the event:
ii) *Recurrence(C*,*S*_*rnd*_*)* ≥ *Recurrence(C*,*S*_*obs*_*)*
  Our null hypothesis is that both events i) and ii) occur by chance. Similar to widely used methods such as GSEA [64], we used permutation testing to establish a null distribution for *S*. We generated 20,000 permutations for each individual cancer site, by keeping the sample pairing (as indicated by the patient id) but randomly assigning the case/control status within each pair. For each permutation S we applied conditional logistic regression for each cancer type, and counted events i) and ii) as described above. We assigned each event a *P* value corresponding to the relative number of permutations (out of 20,000) for which events i) or ii) were observed. Statistical significance was achieved at the FDR < 0.25 level, across the top ten most CpG-rich clusters.

## Additional files

Additional file 1: Supplementary figures. (DOCX 9342 kb)

Additional file 2: Supplementary tables. (XLSX 808 kb)
