# Supplementary material for: Epigenetic supersimilarity of monozygotic twin pairs
Source: Genome Biol. 2018 Jan 9;19:2. doi: 10.1186/s13059-017-1374-0 (PMC5759268; doi:10.1186/s13059-017-1374-0)
Supplement: Supplementary file 1 — Supplementary figures. (DOCX 9342 kb) [file 13059_2017_1374_MOESM1_ESM.docx]

Supplementary Information for

**Epigenetic supersimilarity of monozygotic twin pairs**

Timothy E. Van Baak^1^, Cristian Coarfa^1^, Pierre-Antoine Dugué, Giovanni Fiorito, Eleonora Laritsky, Maria S. Baker, Noah J. Kessler, Jianrong Dong, Jack D. Duryea, Matt J. Silver, Ayden Saffari, Andrew M. Prentice, Sophie Moore, Akram Ghantous, Michael N. Routledge, Yun Yun Gong, Zdenko Herceg, Paolo Vineis, Gianluca Severi, John L. Hopper, Melissa C. Southey, Graham G. Giles, Roger L. Milne, Robert A. Waterland^2^

^1^These authors contributed equally.

^2^Correspondence to: [waterland@bcm.edu](mailto:waterland@bcm.edu) .

**This file includes:**

Figures S1 to S11

**Other Supplementary Material for this manuscript includes:**

Supplementary Tables 1 to 18 (Additional File 2):

ST1 Annotated list of ESS and SIV probes

ST2 Summary of pyrosequencing results on ESS hits

ST3 Summary of pyrosequencing results on SIV hits

ST4 Annotated list of negative control probes

ST5 TCGA abbreviations used in Fig. 4E

ST6 MCCS HM450 data sets, and average years from sample collection to diagnosis

ST7 ESS clusters

ST8 Associations between ESS cluster average methylation in adipose tissue and expression in adipose tissue, LCL, and skin

ST9 SIV clusters

ST10 Negative control clusters

ST11 MCCS permutation testing results: ESS clusters showing significant associations with later cancer

ST12 MCCS permutation testing results: Neg. contr. clusters showing significant associations with later cancer

ST13 MCCS permutation testing results for 10 most CpG-rich ESS clusters

ST14 ESS clusters containing no probes positive for substantial mQTL

ST15 Validation data on pyrosequencing assays

ST16 Season of conception study: principle component analysis results

ST17 Gambian SoC-DMP enrichment analysis without cell composition adjustment

ST18 Gambian SoC-DMP enrichment analysis with adjustment for additional principal component


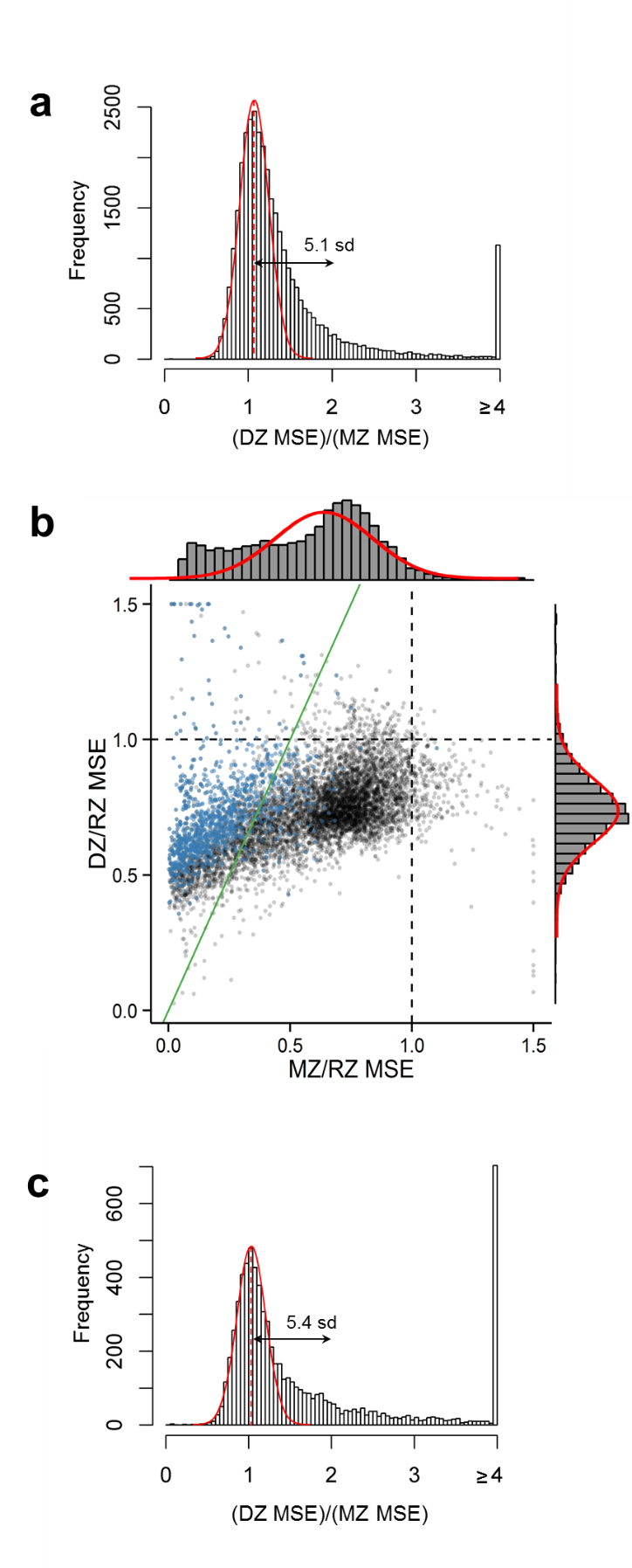


**Figure S1: Statistical significance of ESS probes.** **a**, Unlike the expected normal distribution, the ratio of DZ MSE to MZ MSE is skewed strongly to the right. (ESS probes are selected based on (DZ MSE)/(MZ MSE) > 2.) Relative to a normal distribution modeled on the left side of the data (red, mean ± sd = 1.075 ± 0.18) probability of ESS probes is P < 0.0001. **b**, The same analysis as in Fig. 2b, but including only probes with interindividual β range > 0.4 in the Grundberg et al. data set. Compared to Fig. 2b, the DZ/RZ MSE (right histogram) remains normally distributed, but the left skewing of the MZ/RZ MSE (top histogram) is more pronounced. (Red curves show normal curves fitted to the data.) **c**, DZ MSE to MZ MSE distribution of probes with range > 0.4 in the Grundberg et al. data set. Relative to a normal distribution modeled on the left side of the data (red, mean ± sd = 1.025 ± 0.18) enrichment of ESS probes remains highly significant (P < 0.0001).


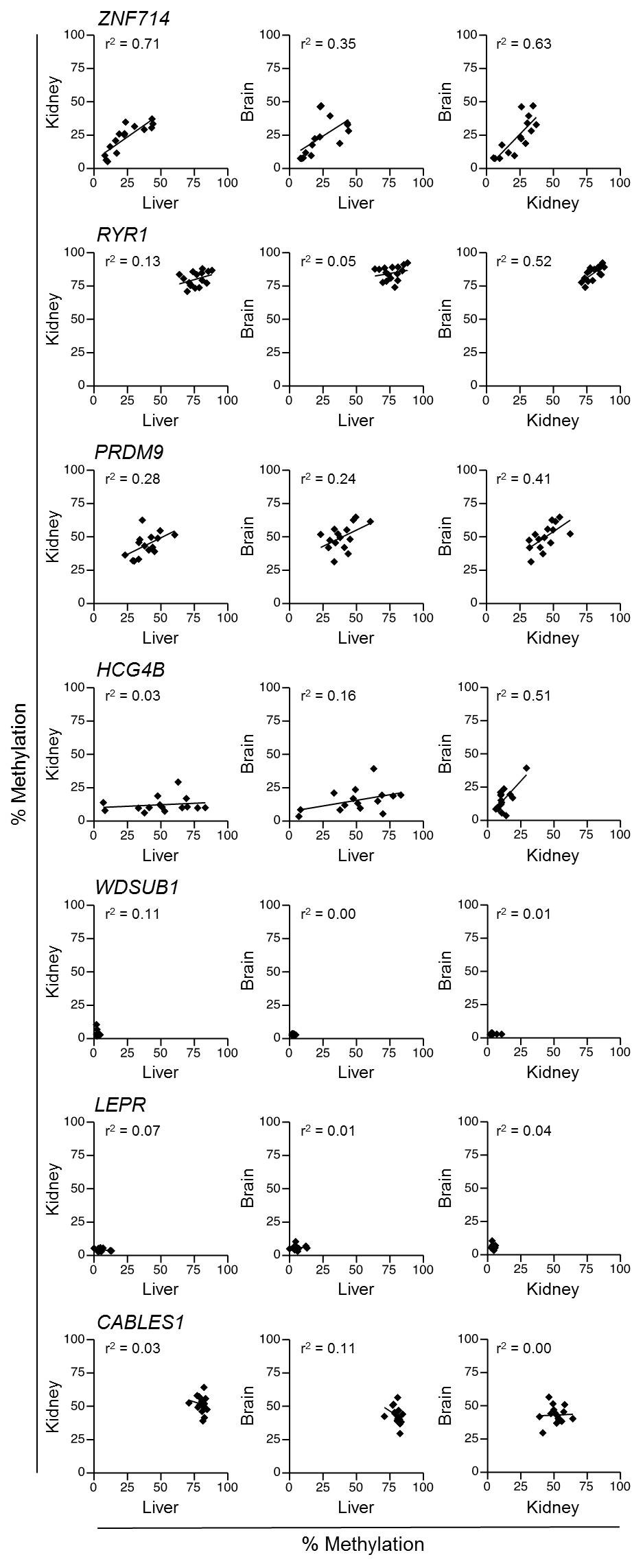


**Figure S2:** **Bisulfite pyrosequencing validation data in ESS hits, to test for SIV.** Each plot shows inter-tissue correlation for tissues from 17 cadavers. *ZNF714, RYR1, PRDM9,* and *HCG4B* yielded at least one inter-tissue correlation with r^2^ > 0.50 (i.e. validated). *WDSUB1, LEPR,* and *CABLES1* failed to validate.


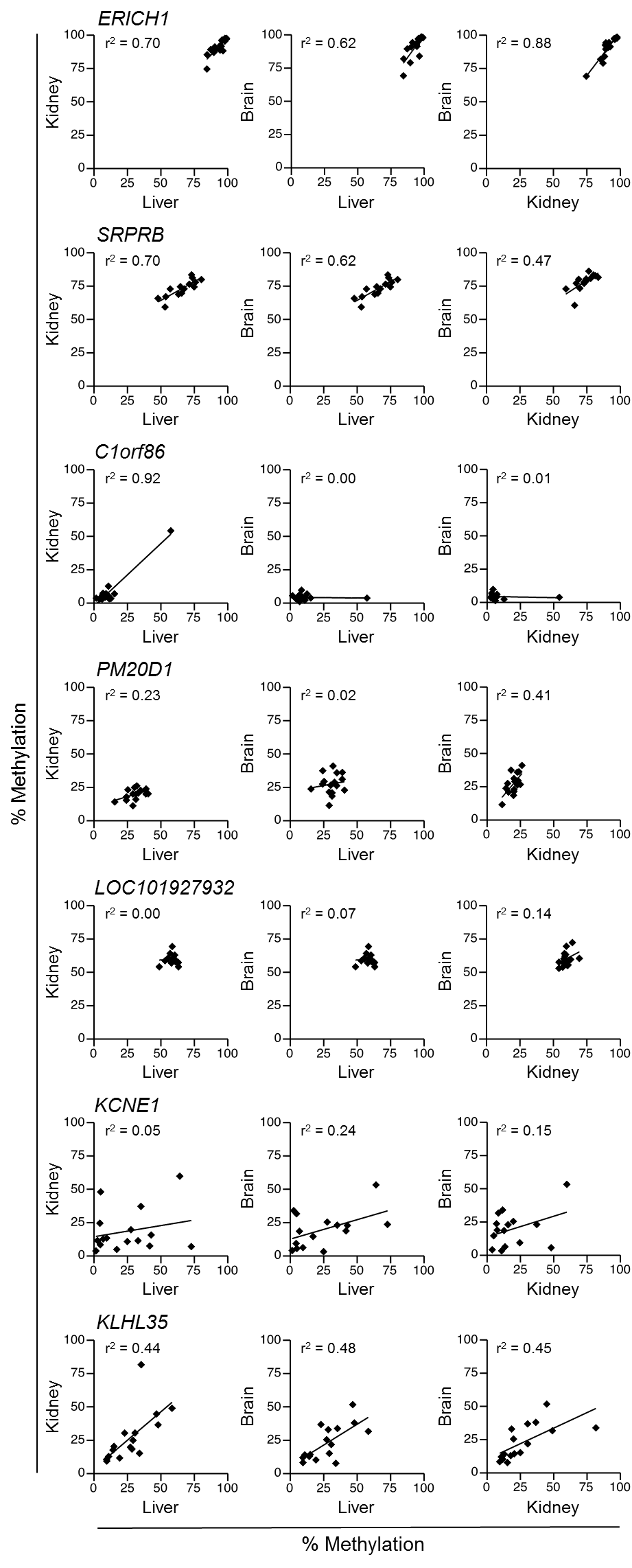


**Figure S3:** **Bisulfite pyrosequencing validation data in hits from the SIV screen.** Each plot shows inter-tissue correlation for tissues from 17 cadavers. *ERICH1*, *SRPRB*, and *C1ORF86* yielded at least one inter-tissue correlation with r^2^ > 0.50 (i.e. validated). *PM20D1*, *LOC101927932*, *KCNE1*, and *KLHL35* failed to validate (although *PM20D1* and *KLHL35* achieved inter-tissue correlations just short of the cutoff).


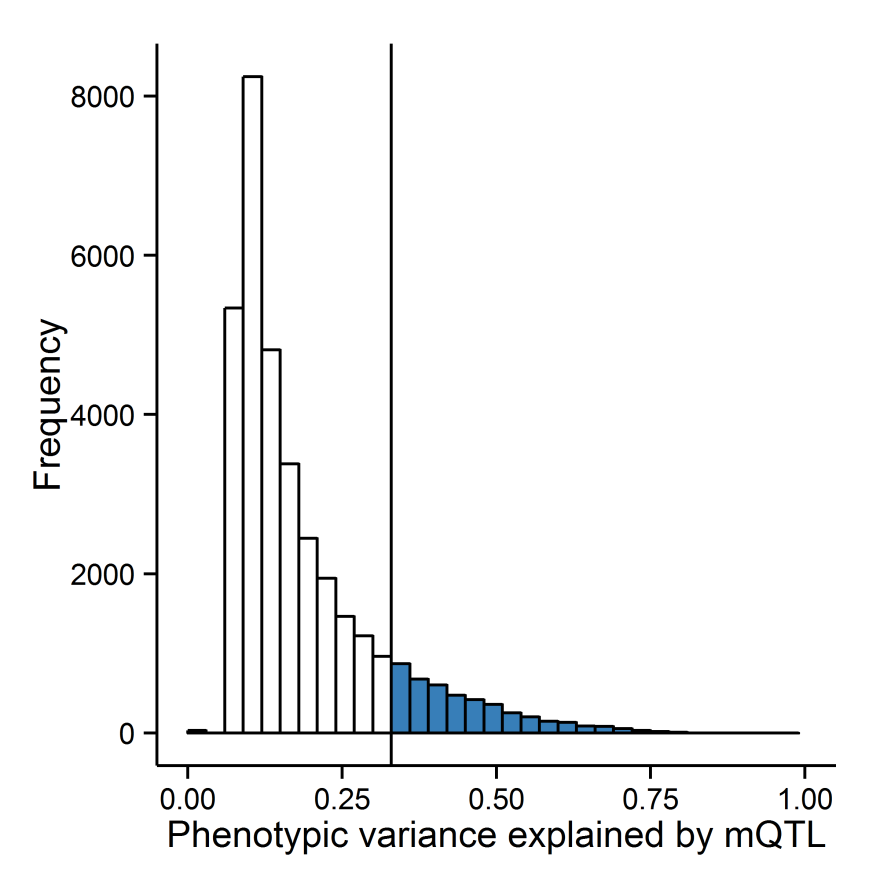


**Figure S4. Cutoff for substantial mQTL.** Of the 34,304 probes Shi et al. identified as showing significant *cis*-mQTL, only 4,306 (blue) have at least 33% of their variation explained by mQTL.


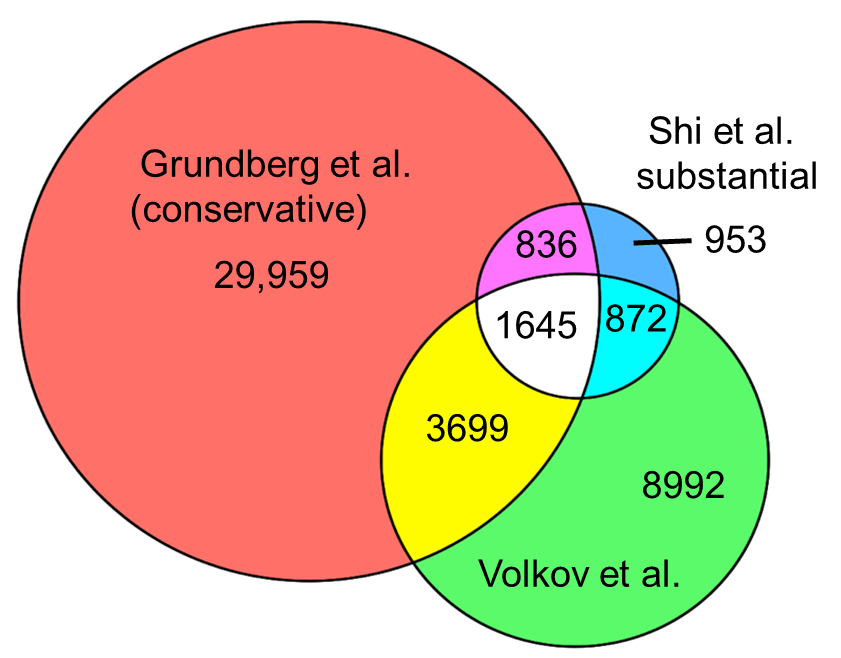


**Figure S5. HM450 probes identified as mQTL in three different studies.** Venn diagram illustrates overlap of probes identified as showing mQTL in Grundberg *et al*. (n=36,139, conservative P value cutoff), Volkov *et al*. (n=15,208), and Shi *et al*. (n=4306, substantial mQTL (i.e. *β_SNP_* > 0.33)). Over half of the Shi *et al*. substantial mQTL probes overlap with either of the other two sets.


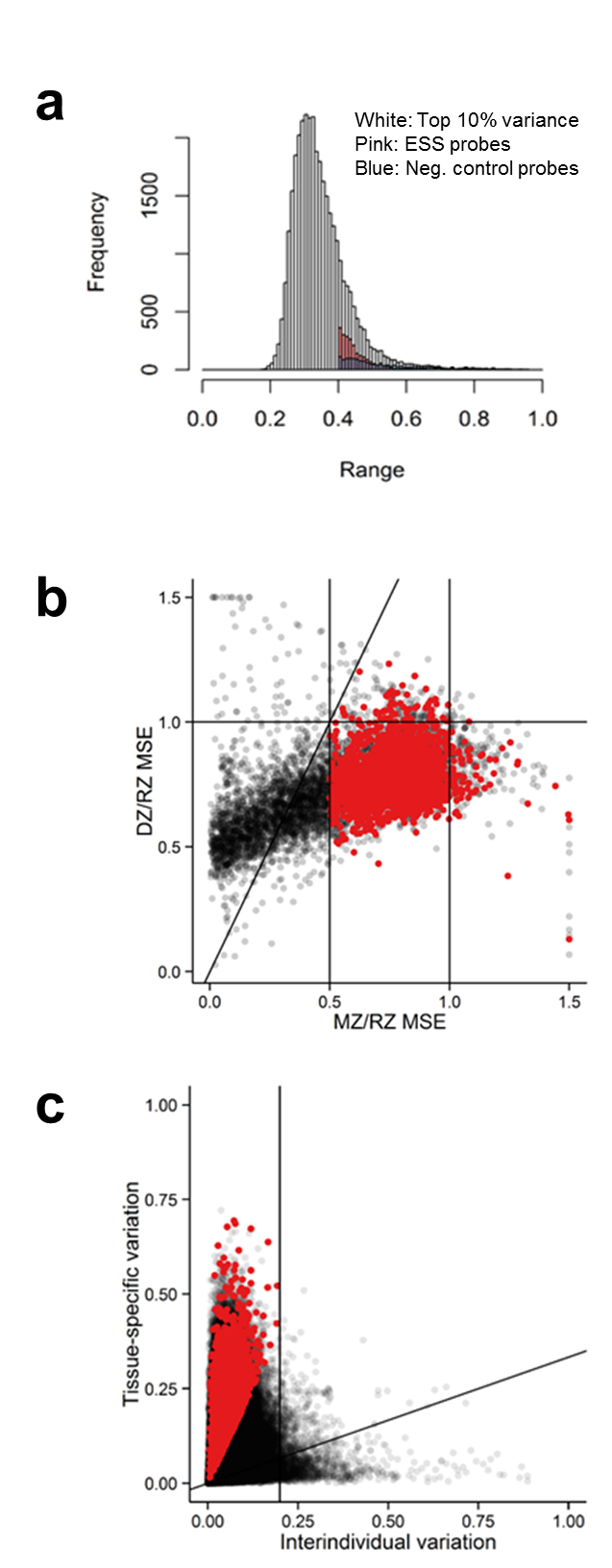


**Figure S6. Negative control probes.** **a**, Negative control probes all have interindividual range >0.4 in the Grundberg data set. **b**, Negative control probes (red dots) compared to Figure 2B; they are distinct from ESS hits. **c**, Negative control probes (red dots) compared to Figure 3B; they are distinct from SIV hits.

**
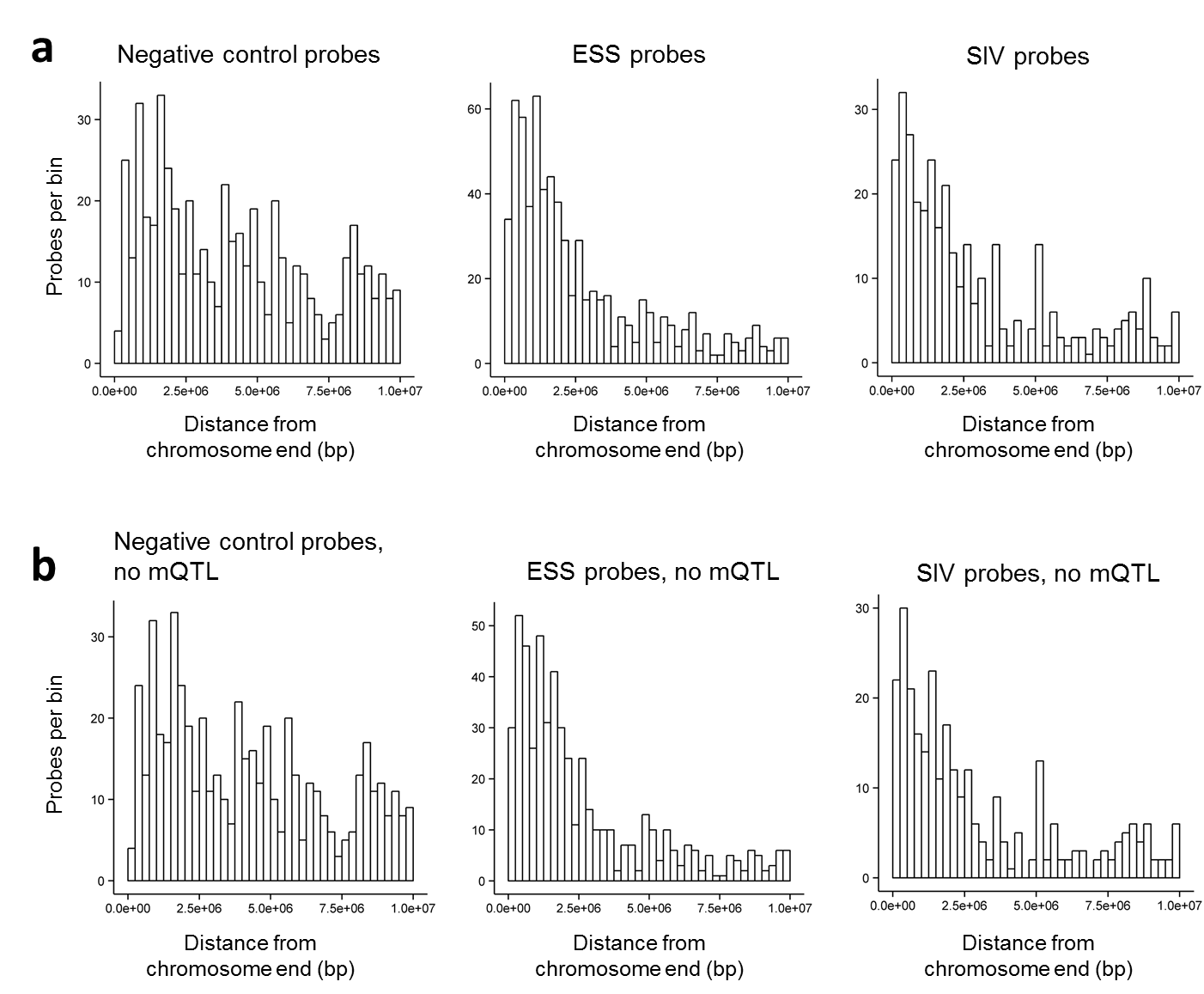
**

**Figure S7. Both ESS and SIV probe sets are enriched in subtelomeric regions. a**, Distribution of ESS and SIV probes in the 10 Mb from all chromosome ends, relative to negative control probes. Both ESS and SIV probes are concentrated in the 2 Mb subtelomeric regions. **b**, Same plots, but excluding probes with evidence of substantial mQTL. The same enrichment is observed, indicating that the subtelomeric enrichment is not due to the greater concentration of SNPs (and hence greater potential for mQTL) in subtelomeric regions.


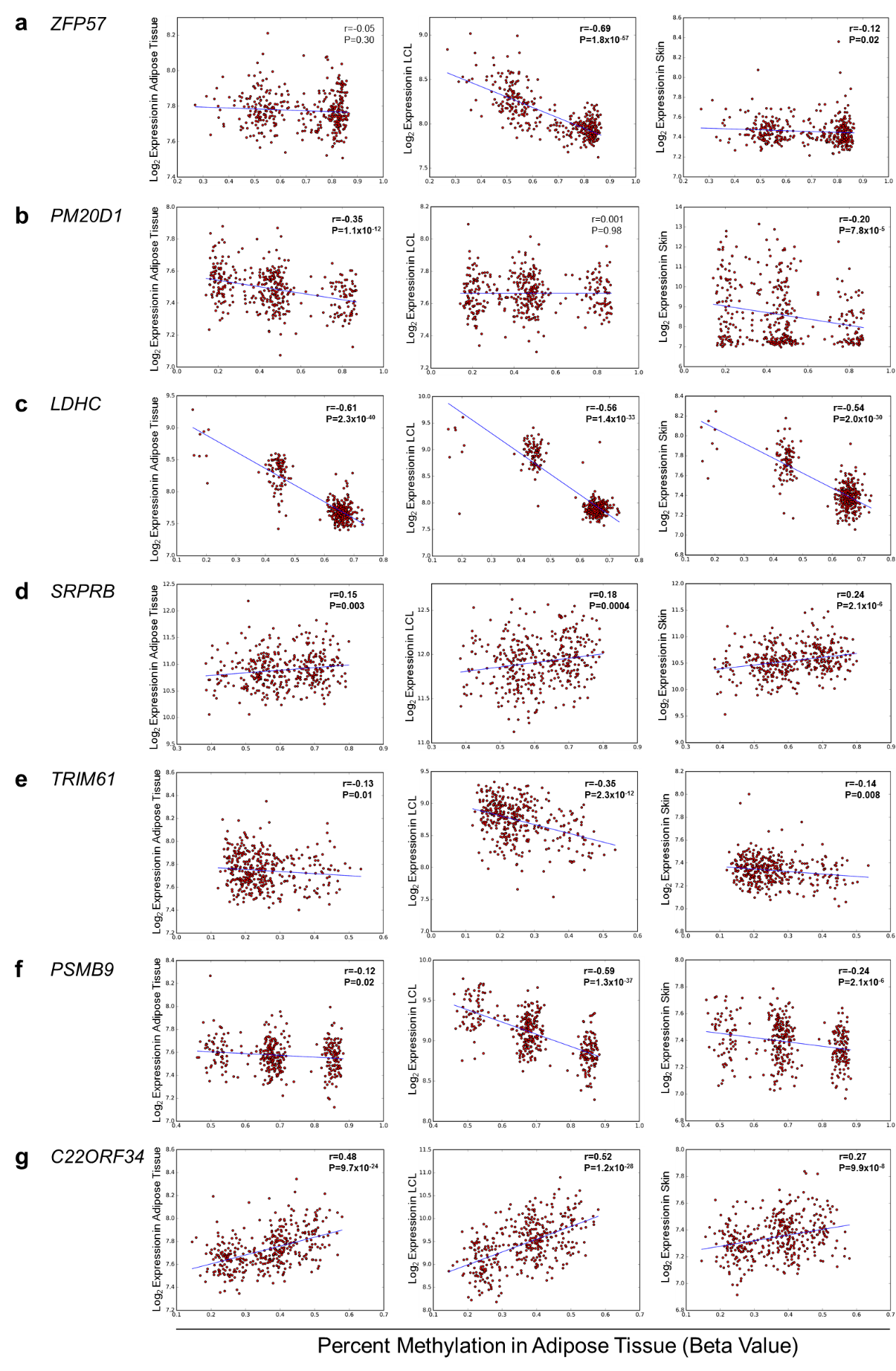


**Figure S8. Examples of associations between cluster-average methylation in adipose tissue and expression of associated genes in adipose tissue (left), lymphoblastoid cell lines (LCL, middle), and skin (right).** Associations are illustrated for **a**, *ZFP57*, **b**, *PM20D1*, **c**, *LDHC*, **d**, *SRPRB*, **e**, *TRIM61*, **f**, *PSMB9*, and **g**, *C22ORF34*. At *ZFP57*, methylation in adipose tissue is inversely associated with expression in LCL but not in adipose tissue or skin. At *LDHC*, methylation in adipose tissue is strongly inversely associated with expression in adipose tissue, LCL, and skin. At *SRPRB* and *C22ORF34*, conversely, methylation in adipose tissue is positively associated with expression in adipose tissue, LCL, and skin.


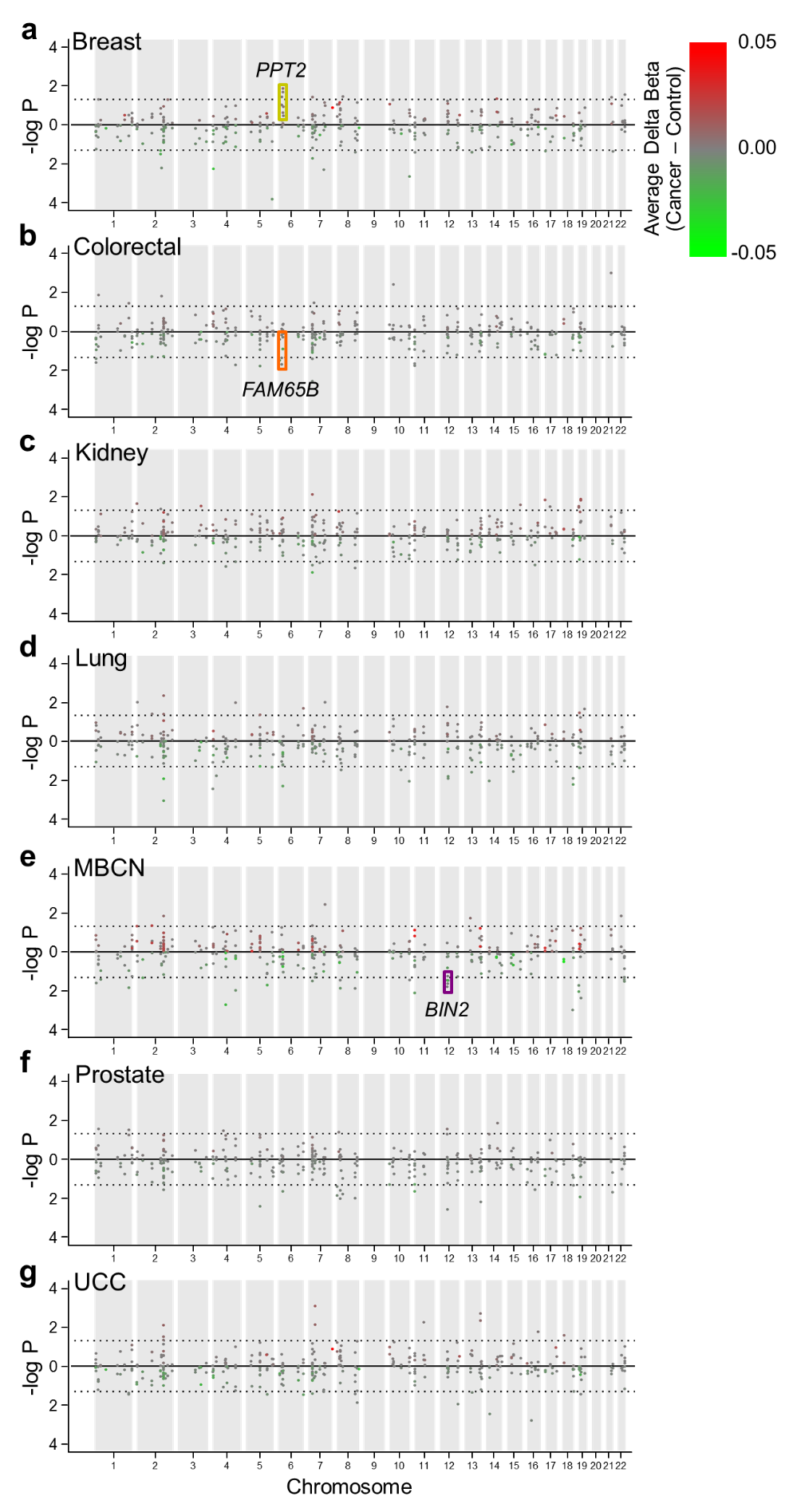


**Figure S9. At clusters of negative control probes, peripheral blood methylation at baseline is not strongly associated with risk of later cancer.** Associations are tested with respect to **a**, Breast cancer, **b**, Colorectal cancer, **c**, Kidney cancer, **d**, Lung cancer, **e**, Mature B cell neoplasm, **f**, Prostate cancer, and **g**, Urothelial cell carcinoma. Unlike at the ESS clusters (Fig. 7), methylation is significantly associated with risk of later cancer at only a few of the top 13 most CpG-rich negative control clusters (FDR<0.25, labeled boxes). In every case, the magnitude of the effect is extremely small (Delta Beta < 0.01) (Table S12).


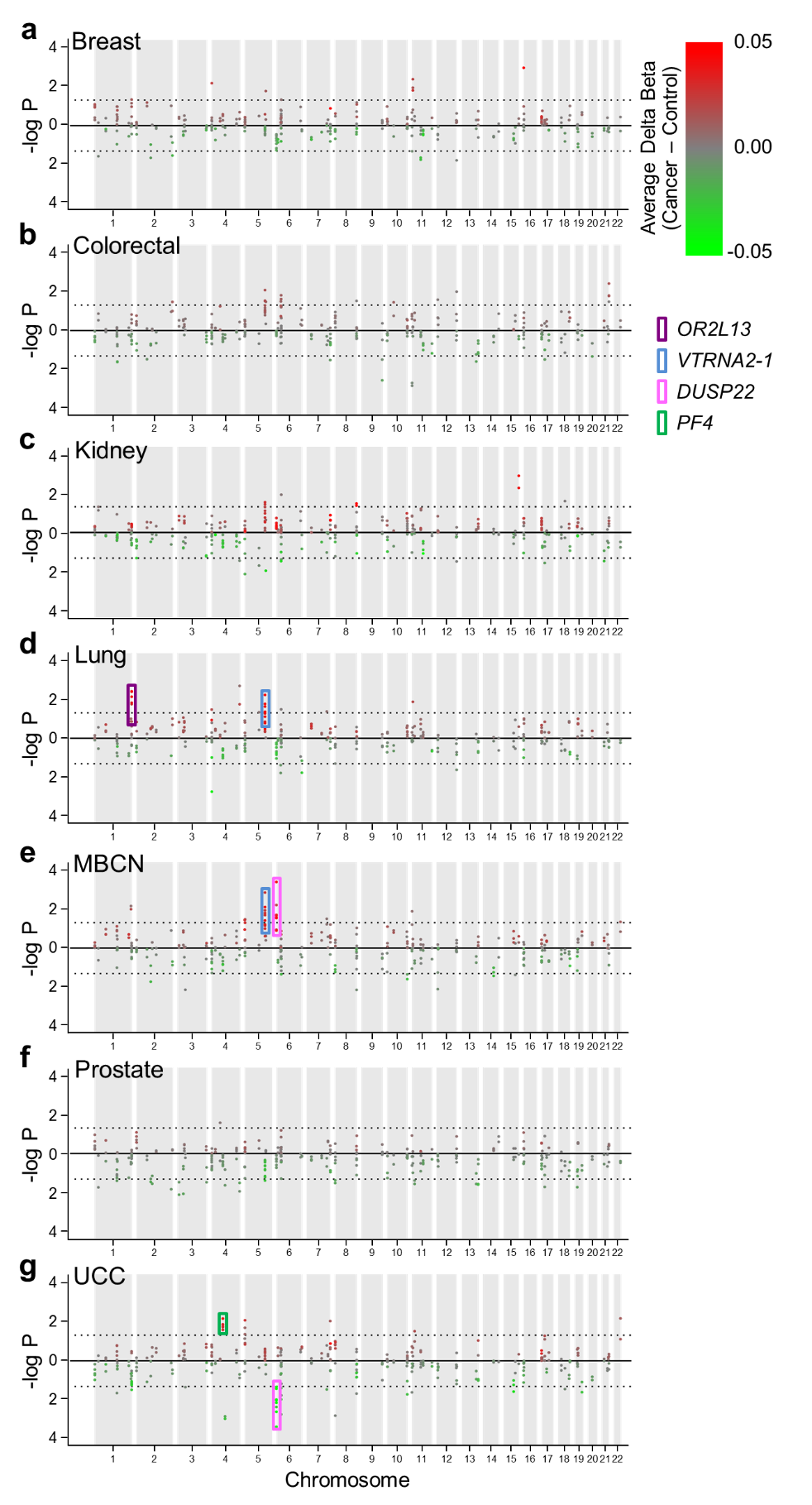


**Figure S10. Associations with later cancer among those of the top 10 ESS clusters containing no substantial mQTL probes.** Associations are tested with respect to **a**, Breast cancer, **b**, Colorectal cancer, **c**, Kidney cancer, **d**, Lung cancer, **e**, Mature B cell neoplasm, **f**, Prostate cancer, and **g**, Urothelial cell carcinoma. Of the 10 significant associations illustrated in Figure 7, 6 are at ESS clusters with no evidence of mQTL.


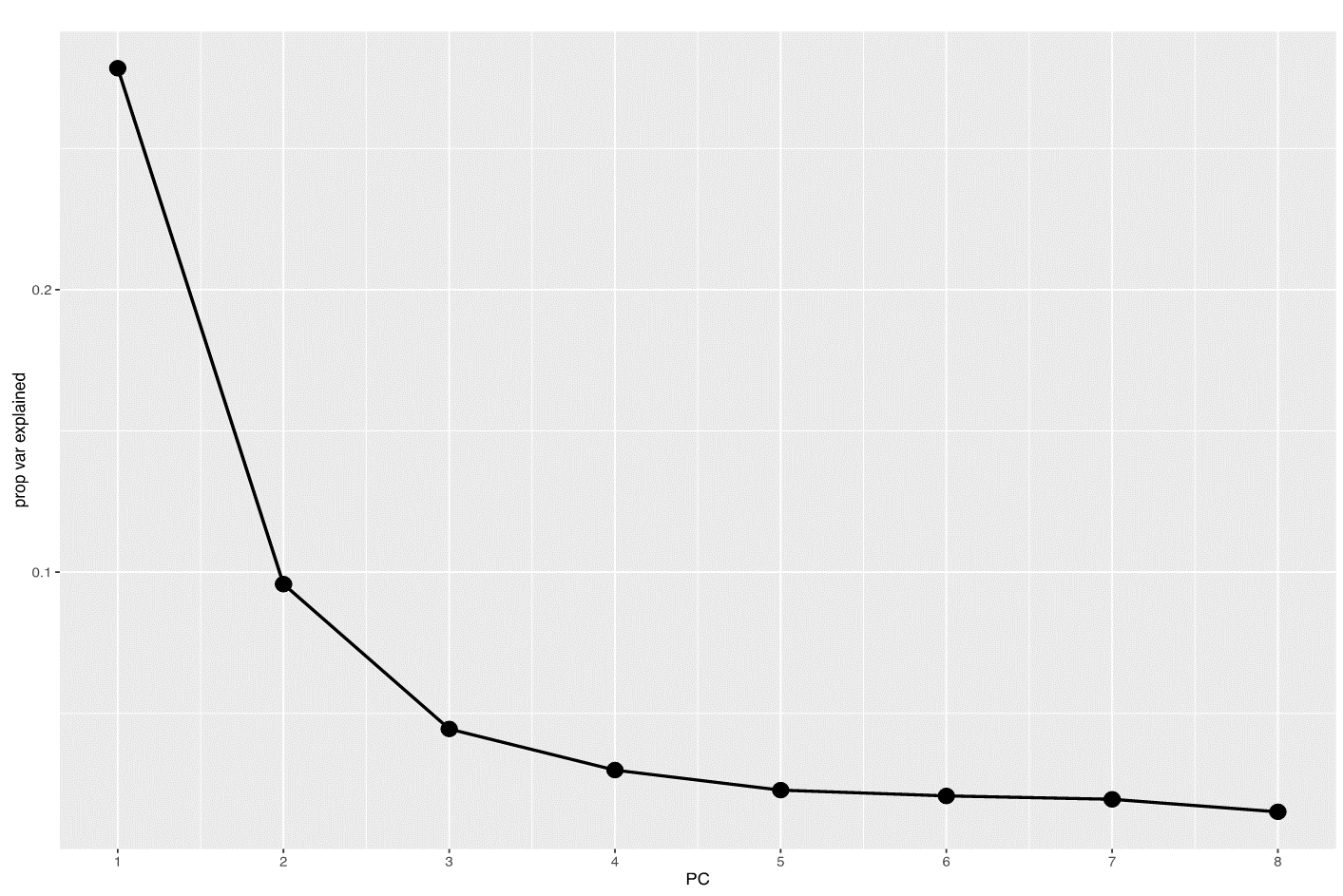


**Figure S11. Season of conception study: Principal component analysis scree plot**
